# Targeted autophagic clearance of Tau protects against Alzheimer's disease through amelioration of Tau-mediated lysosomal stress

**DOI:** 10.7150/thno.118409

**Published:** 2025-08-16

**Authors:** Bo Hyun Yoon, Jinho Kim, Sandip Sengupta, Chan-Jung Park, Minjoo Ko, Ji Hee Kang, Young Tag Ko, Yeji Kim, Seung Min Lim, Yoonhee Bae, MooYoung Choi, Yunyeong Jang, Ho Jeong Kwon, Hyo Jin Son, Hee Jin Kim, Taebo Sim, Keun-A Chang, Myung-Shik Lee

**Affiliations:** 1 Soonchunhyang Institute of Medi-bio Science, Department of Internal Medicine, Soonchunhyang University College of Medicine, Cheonan, Korea.; 2 Department of Biomedical Sciences, Yonsei University College of Medicine, Seoul, Korea.; 3 Dalim Fromtech, Inc., Seoul, Korea.; 4 Dept. of Pharmacology, College of Medicine, GAIHST, Gachon University, Incheon, Korea.; 5 Dept. of Health Sciences and Technology, GAIHST, Gachon University, Incheon, Korea.; 6 Graduate School of Clinical Drug Discovery & Development, Yonsei University College of Medicine, Seoul, Korea.; 7 Graduate School of Medical Science, Brain Korea 21 Project, Yonsei University College of Medicine, Seoul, Korea.; 8 Clinical Candidate Discovery & Development Institute, Yonsei University College of Medicine, Seoul, Korea.; 9 College of Pharmacy and Gachon Institute of Pharmaceutical Sciences, Gachon University, Incheon, Korea.; 10 Department of Physics and Center for Theoretical Physics, Seoul National University, Seoul, Korea.; 11 Chemical Genomics Leader Research Laboratory and Department of Biotechnology, College of Life Science & Biotechnology, Yonsei University, Seoul, Korea.; 12 Dept. of Neurology, Samsung Medical Center, Sungkyunkwan University School of Medicine, Seoul, Korea.; 13 Division of Endocrinology, Department of Internal Medicine, Soonchunhyang University College of Medicine, Cheonan, Korea.; The first 3 authors contributed equally to this work.

**Keywords:** Tau, Alzheimer's disease, lysosomal stress, autophagy, specificity

## Abstract

**Background:** Lysosomal dysfunction could be an underlying cause of Alzheimer's disease, with Tau oligomer being an important inducer or amplifier of lysosomal stress associated with the disease. Tau oligomer is a well-known substrate of autophagy, and selective degradation of Tau with Tau-specific autophagy degrader might be feasible.

**Methods:** Tau-specific autophagic degraders were synthesized by combining leucomethylene blue, linkers and a lysosomal degradation tag (Autac). Tau clearance and changes of Tau-mediated lysosomal stress by these degraders were studied *in vitro*. *In vivo* effects of a Tau-specific degrader were investigated employing a combined Tau/Aβ mutant mouse model characterized by an accelerated onset of neurological deficits. Human relevance was investigated using induced pluripotent stem cell (iPSC)-derived neuronal cells from an Alzheimer's disease patient.

**Results**: Among Tau-specific Autac degraders, TauAutac-3 (TA-3) efficiently degraded Tau oligomer and monomer, an effect inhibited by bafilomycin A1, suggesting lysosomal Tau degradation. TA-3 treatment induced LC3, K63, OPTN or NDP52 puncta, which was partially colocalized with Tau oligomer. Signs of lysosomal stress, such as galectin-3 puncta, pHluorin fluorescence, altered lysosomal pH and CHMP2B recruitment, induced by Tau expression were reversed by TA-3. Autophagy impairment by Tau expression *in vitro*, likely due to lysosomal stress, was also reversed by TA-3. *In vivo*, TA-3 administration markedly reduced the accumulation of both Tau and Aβ in 6xTg mice, which was associated with amelioration of Tau-mediated lysosomal stress and autophagy impairment. Neuroinflammation characterized by increased numbers of GFAP^+^ glial cells and Iba1^+^ microglial cells, was also reduced following TA-3 administration. TA-3 remarkably improved neurologic deficits in 6xTg mice, such as impaired memory and reduced exploratory behavior. TA-3 reduced Tau and phospho-Tau accumulation in iPSC-derived neuronal cells from an Alzheimer's disease patient.

**Conclusion:** These results suggest that Tau-specific autophagic (Autac) degraders could serve as novel therapeutic agents for Alzheimer's disease through reduction of Tau-mediated lysosomal stress.

## Introduction

Alzheimer's disease (AD) is the most prevalent and important member of debilitating neurodegenerative diseases characterized by the accumulation of brain amyloid plaques or neurofibrillary tangles (NFTs) and progressive cognitive decline or memory loss 1. The main constituents of brain amyloid plaques and NFTs are Aβ and Tau aggregates, respectively 2. Previous studies have focused on specific Aβ clearance, based on the hypothesis that amyloid plaque accumulation is a fundamental mechanism of AD and that removal of Aβ oligomer or fibril could halt or reverse the disease 2. Antibody-based therapies targeting of Aβ such as Aducanumab and Lecanemab, have been approved by the FDA and could slow cognitive deterioration 3, 4. However, their clinical effects are predominantly observed in early AD with mild cognitive impairment, and their use has been associated with adverse effects including brain hemorrhage or edema, warranting further studies on the efficacy and safety of anti-Aβ antibodies 4. In addition to Aβ, Tau has emerged as an important target for intervention 5. Indeed, genetic deletion of *Tau* alone or immunization against Tau has been shown to ameliorate clinical and pathological abnormalities in AD animal models, highlighting the critical role of Tau in AD pathogenesis 6. Clinically, imaging of Tau NFT can provide a useful tool assessing the progression of neurodegeneration and the extent of disease spreading, which is not the case of Aβ imaging 7, 8. Furthermore, recent studies have implicated lysosomal dysfunction as a crucial factor in AD pathogenesis 9, with Tau playing a central role in lysosomal damage associated with the disease 10.

Protein degradation primarily occurs through two pathways: the ubiquitin-proteasomal degradation and autophagy-lysosomal pathways. Soluble proteins can be degraded by both mechanisms, however; aggregate or amyloidogenic proteins are preferentially cleared by autophagy-lysosomal pathway 11. Given that most pathogenic proteins implicated in neurodegenerative diseases such as AD, Parkinson's disease or amyotrophic lateral sclerosis are aggregate-prone or amyloidogenic 12, autophagy-lysosomal pathway is pivotal in their clearance. In AD, both Aβ and Tau proteins are well-documented aggregate-prone or amyloidogenic proteins 2. While proteasomal pathway also contribute to Aβ and Tau clearance, autophagy plays indispensable roles in degrading critical pathogenic forms 13. For example, acetylated Tau and hyperphosphorylated paired helical filament (PHF) form of Tau both of which are important elements in AD progression, are primarily cleared by autophagy 14, 15. Similarly, caspase-cleaved Tau which has an enhanced ability to form self oligomers, is also cleared by autophagy 16.

Efforts to accelerate autophagy-lysosomal clearance of Aβ or Tau oligomer have included novel agents or drug repositioning strategies. Although most approaches have aimed at enhancing bulk autophagy-lysosomal degradation pathway, recent studies have attempted Tau clearance using specific autophagic or proteasomal degraders 17, 18. In this study, we developed a specific Tau degrader comprising a Tau-targeting warhead, a linker and a lysosomal degradation tag (Autac) which efficiently cleared Tau *in vitro* and *in vivo* in association with reduction of Tau-mediated lysosomal stress and restoration of impaired autophagic activity. These improvements led to amelioration of neurological deficits, accompanied by clearance of Aβ in mice with combined Tau and Aβ accumulation 19. Accumulation of Tau and phospho-Tau in iPSC-derived neuronal cells from a sporadic AD patient was also alleviated by the Tau-specific degrader, suggesting therapeutic potential against human AD.

## Methods

### Animals

6xTg mice were generated by crossbreeding 5xFAD (B6SJL) transgenic (Tg) mouse (Jackson Laboratory, Bar Harbor, ME, USA) with JNPL3 Tau(P301L) Tg mouse (Taconic Biosciences Inc., Albany, NY, USA), as previously described 19, and were genotyped by PCR using genomic DNA from ear biopsy samples. Male wild-type (WT) and JNPL3^+/-^5xFAD^+/-^ (6xTg) mice were housed on a 12-h light/dark cycle with *ad libitum* access to food and water in an automatically controlled environment at 22 ± 2 °C and 50 ± 10% relative humidity. All animal experiments were performed in compliance with the Animal Care and Use Guidelines of the Gachon University (IACUC No: LCDI-2021-0171).

### Synthesis of TauAutac (TA)

See Supplementary Methods.

### Pharmacokinetic study and LC-MS/MS

See Supplementary Methods.

### Drug treatment *in vivo*

TA-3 was dissolved in distilled water (DW) containing 5% NMP, 5% polyethylene glycol and 5% solutol solution that have been used as excipients or solvents for human use or in human trials 20, 21, 22. DW containing 5% NMP, 5% polyethylene glycol, and 5% solutol was used as a vehicle. Four-month-old 6xTg or WT mice were injected intraperitoneally (i.p.) with 5 mg/kg of TA-3 daily (six times/week) for two months. The number of mice per each experiment was estimated based on our previous experience with 6xTg mice 19, and was adjusted in accordance with the 3R (Reduction, Replacement and Refinement) animal research guidelines 23, recommended by IACUC of Gachon University.

### Behavior test

Neurological evaluation was conducted as previously described, with modifications 19. Three behavioral tests were performed to assess changes in memory and cognition after TA-3 treatment. All tests were automatically recorded and analyzed using the Ethovision XT 9 system (Noldus Information Technology, Wageningen, the Netherlands). For the novel object recognition (NOR) test, mice were habituated to an open field chamber (38 cm wide x 38 cm high x 40 cm long) for 20 min on the first day. On the following day, mice were placed in the same open chamber and exposed to two identical objects for five min. The objects (height, 10 ± 2 cm) were colored and filled with stones to prevent displacement by experimental animals. They were positioned five cm away from the walls of the box in opposite corners. After 24 h, one familiar object was replaced with a novel object of different colors and shapes, and the exploration time in areas containing the familiar or novel object was determined for five min. Memory index was calculated as the percentage of exploration time for novel object divided by the total exploration time. The Y-maze test was performed using a white polyvinyl plastic maze with 3 branches of 40 cm in length, 6.8 cm wide and 15.5 cm high. Mice were placed in the maze for eight min, and the consecutive entries into three different arms was given one point (alternation). Spontaneous alternation was calculated using the formula: (number of alternations/[total entries-2] x 100(%) 19. The passive avoidance test was performed over three continuous days using an avoidance learning box (Gemini Passive Avoidance System, San Diego Instruments, San Diego, CA, USA) consisting of adjacent bright and dark chambers connected by a remote operational gate. The bright chamber was illuminated with a fixed W LED light. On the first day, mice were allowed to explore the chambers freely. On the next day, mice that entered the dark chamber were given an electric shock to the feet (0.3 mA for three sec). After 24 h, latency time before entering the dark chamber was recorded.

### Tissue preparation

Mice were anesthetized with a mixture of Zoletil (8.3 mg/kg) and Rompun (15 mg/kg), and then the brains were harvested. The cortex and hippocampus were dissected from one hemisphere and immediately frozen in liquid nitrogen for immunoblotting. The other hemisphere of each mouse brain was fixed in 4% paraformaldehyde at 4 °C for 24 h, then dehydrated in a 30% sucrose solution for three days. Dehydrated tissues were frozen in molds filled with optimal cutting temperature compound (Sakura, Osaka, Japan). Frozen tissues were cut at a thickness of 30 μm using a cryomicrotome (Thermo Electron Corporation, Waltham, MA, USA), and stored in a cryoprotectant solution (30% ethylene and 30% glycerol in PBS) at 4 °C until use.

### Tissue staining

For immunohistochemistry, 30 μm sections were incubated in 3% H_2_O_2_ for 20 min, followed by washing in PBS for 10 min. After blocking with 0.5% BSA-3% normal goat serum in 0.4% PBS-T for 1 h, slices were incubated with a primary antibody at 4℃ overnight. The next day, sections were incubated with a secondary antibody and then with ABC kit (Vector Laboratories, Newark, CA, USA), followed by color development with DAB solution (Abcam, Cambridge, UK). Mounted sections were observed under a light microscope (Zeiss, Oberkochen, Germany), and quantified using ImageJ software. Immunofluorescence staining was performed as previously described with modifications 19. Briefly, brain tissues were washed with 0.2% Triton X-100 in PBS, blocked with 1% BSA-3% normal goat serum in 0.4% PBS-T at room temperature (RT) for 1 h, and incubated with primary antibodies [AT8 (Thermo Fisher, Waltham, MA, USA), 6E10 (Thermo Fisher), anti-LAMP1 (Abcam), anti-LC3 (Novus Biologicals, Centennial, CO, USA)] in PBS-T at 4 °C overnight. The following day, sections were incubated with Alexa 488- or Alexa 555-conjugated secondary antibodies (Invitrogen) at RT for 1 h. Amyloid plaque staining was performed by incubation in Thioflavin-S (Sigma-Aldrich) solution at RT for 8 min. Images were captured using a Nikon TS2-S-SM microscope (Nikon Microscopy, Tokyo, Japan) equipped with a Nikon DS-Qi2 camera. Fluorescence intensity in the regions of interest was quantified. For analysis of the brain images, three sections per mouse were stained and one field per mouse section was studied in a blinded manner.

### SA-β-gal staining

Cells prefixed with 2% formaldehyde/0.2% glutaraldehyde were incubated with SA-β-Gal staining solution comprising 1 mg/mL X-Gal, 40 mM citric acid/sodium phosphate, pH 6.0, 5 mM potassium ferrocyanide, 5 mM potassium ferricyanide, 150 mM NaCl and 2 mM MgCl_2_. After the full development of color reaction, cells were visualized under a light microscope.

### Transfection

T-REx-*Tau40*-HEK293 cells that were generated by transfecting HEK293 cells with T-REx-*Tau40* (kindly provided by Min Jae Lee, Seoul National University) 24 or T-REx-*mTRPM7*-HEK293 cells 25 were transiently transfected with the following plasmids: 0.5 μg of *pmCherry-Gal3* (Addgene), *mRFP-EGFP-LC3* (kindly provided by Tamotsu Yoshimori, Osaka University) or *CD63-pHluorin* (Addgene), using Lipofectamine 2000 (Invitrogen, Carlsbad, CA, USA), according to the manufacturer`s protocol.

### Cell treatment and staining

To study effect of test agents on Tau clearance, T-REx-*Tau40*-HEK293 cells were treated with 500 pg/mL of doxycycline for 24 h, and then with test chemicals without doxycycline for another 24 h, followed by immunoblot analysis. To examine the induction of autophagy and recruitment of autophagy receptors or ubiquitin by TA, double immunofluorescence staining was conducted using the following antibodies: anti-LC3 (Novus Biologicals), -K63 (Millipore, Burlington, MA, USA), -K48 (Santa Cruz, Dallas, TX, USA), -p62 (Progen, Wayne, PA, USA), -NDP52 (Abnova, Walnut, CA, USA) or -OPTN (Santa Cruz) antibody together with TOMA-1 (for K63 costaining) (Merck, Kenilworth, NJ, USA) or T22 antibody (Merck) (for all other costaining) specific for Tau oligomer 26, 27. After further incubation with Alexa 488-anti-IgG (anti-mouse IgG for TOMA-1 or -rabbit IgG for T22 antibody) and Alexa 594-anti-IgG (Invitrogen), LC3, K63, K48, p62, NDP52 and OPTN puncta colocalized with oligomeric Tau were manually identified and quantified in a blinded manner by confocal microscopy (Zeiss LSM780). Clusters of pixels displaying brighter fluorescence than the surrounding ones and appearing obviously larger than the LAMP1 signal in unstressed cells were considered puncta. Numbers of total microscopic fields analyzed by immunofluorescence staining from more than 3 independent experiments were summed and denoted as *n* in the figure legends. At least >10 cells per microscopic field were analyzed (mostly 40~50 cells). Graphs were drawn using GraphPad Prism 9.1.0 software (GraphPad Software Inc., San Diego, CA, USA).

### Immunoblot analysis

RIPA buffer (150 mM NaCl, 1% NP-40, 0.5% sodium deoxycholate, 0.1% SDS, 50 mM Tris, pH 8.0) containing protease inhibitors (Roche Applied Science, Mannheim, Germany) and a cocktail of phosphatase inhibitors (Sigma-Aldrich) was added to cells or brain samples, and lysates were homogenized on ice for 30 min and centrifuged at 20,000 x *g* for 10 min to obtain the supernatant as the RIPA-soluble fraction. Remaining pellets were washed once with RIPA buffer and then resuspended in insoluble fraction buffer (1 M sucrose, 2% SDS in RIPA buffer), followed by incubation at RT for 1 h. After centrifugation at 20,000 x *g* for 3 min, the supernatant was used as the RIPA-insoluble fraction. Whole cell fraction was prepared using 10 mM Tris-HCl (pH 7.5), 100 mM NaCl, 1% NP-40 lysis buffer containing protease inhibitor/phosphatase inhibitor (Gendepot, Baker, TX, USA). Fractionated or whole cell samples were stored at -80 °C until use. Proteins were quantified using the BCA protein assay kit (Thermo Fisher).

For electrophoretic separation, sample buffer with (for visualization of total Tau or all other proteins) or without β-mercaptoethanol (for visualization of oligomeric and monomeric Tau) was added to the supernatant. Whole cell lysates were subjected to electrophoresis on a 10~20% Novex gel (Invitrogen) or 4~12% Bis-Tris gradient NUPAGE gel (Invitrogen). Fractionated samples were loaded onto an 8 or 12% SDS-PAGE gel. Proteins were transferred onto a polyvinylidene difluoride membrane (Merck). After blocking the membranes with 5% skim milk or 3% BSA in TBS-T at RT for 1 h, membranes were incubated with appropriate primary antibodies (Tau-5 [Thermo Fisher AH50042], Tau-13 [BioLegend 835201, San Diego, USA], AT8, AT180 [Thermo Fisher], AT270 [Thermo Fisher], pS396 [Thermo Fisher], anti-LC3, -Beclin 1 [Cell Signaling, Danvers, MA, USA], -p62, -GAPDH [Santa Cruz] or -ATG7 [Cell Signaling]) at 4°C overnight. After three washes with TBS-T, membranes were incubated with appropriate secondary antibodies at RT for one h. Protein bands were visualized using an enhanced chemiluminescence (ECL) kit (ELPISBIO, Daejeon, Korea), and band intensity was quantified using ImageJ software. For some experiments, staining with Ponceau S (Thermo Fisher) capable of binding to hydrophobic protein region 28 was conducted to visualize protein bands in the insoluble fraction. Numbers of independent experiments of the immunoblot analysis were denoted as *n* in the figure legends.

### Gene knockdown (KD) and RT-PCR

T-REx-*Tau40*-HEK293 cells were transfected with *NDP52* siRNA (Santa Cruz, sc-93738), *OPTN* siRNA (Santa Cruz, sc-39054), si*ATG7* #1 (AUCAGGCACUGCUCUUGAA), si*ATG7* #2 (GCACUAGAGUGUGCAUAUG), si*ATG7* #3 (CAGUGGAUCUAAAUCUCAAACUGAU) (CosmogeneTech, Seoul, Korea) or control siRNA using Lipofectamine RNAiMAX (Invitrogen), according to the manufacturer`s protocol. Efficacy of *NDP52* or *OPTN* siRNA KD was evaluated by RT-PCR. The primer sequences are as follows: *NDP52* forward, 5'-TCACCCAGCATTTCATCCCTC-3'; *NDP52* reverse 5'-GTCC TTGGCTCCTCCATTTG-3'; *OPTN* forward, 5'-GAGAAGGCTCTGGCTTCCAA-3'; *OPTN* reverse, 5'-GTCATGGTTTCCAGG TCCTCT-3'. Expression of *Tau* was evaluated by RT-PCR using primers specific for human *Tau* (forward, 5'-AAGAGGAGTTGAGAGTTCCG-3'; reverse, 5'-CGGCCCAGAGACCCCAGA-3').

### Autophagic activity and lysosomal stress *in vitro*

Autophagic activity was assessed after treatment of *mRFP-EGFP-LC3*-transfected T-REx-*Tau40*-HEK293 with doxycycline for 72 h, followed by treatment with TA-3 for 24 h without doxycycline. Confocal microscopy was conducted to count the numbers of yellow (autophagosome) and red puncta (autolysosome). Autophagic activity in cultured cells was also visualized by immunofluorescence staining using anti-LC3 and anti-LAMP2 (Abcam) antibodies as the primary antibodies, and LC3 puncta colocalized with LAMP2 were quantified 29.

Cellular lysosomal stress was evaluated by the following methods: 1) Galectin-3 puncta assay: *pmCherry-Gal3*-transfected T-REx-*Tau40*-HEK293 or T-REx-*mTRPM7*-HEK293 cells were treated with 500 pg/mL doxycycline for 24 h and then with 10 nM TA-3 for 24 h in the absence of doxycycline. After immunofluorescence staining using anti-LAMP1 antibody as the primary antibody and Alexa488-anti-mouse IgG as the secondary antibody, followed by DAPI staining, Galectin-3 puncta colocalized with LAMP1 were counted by confocal microscopy. 2) pHluorin fluorescence: *CD63-pHluorin*-transfected T-REx-*Tau40*-HEK293 or T-REx-*mTRPM7*-HEK293 cells were treated with 500 pg/mL doxycycline for 24 h and then with 10 nM TA-3 for 24 h in the absence of doxycycline. pHluorin fluorescence was quantified by confocal microscopy. 3) Lysosomal pH changes: Doxycycline-treated T-REx-*Tau40*-HEK293 cells were incubated with 10 nM TA-3 for 24 h in the absence of doxycycline, followed by incubation with 100 μg/mL LysoSensor DND-189 at 37 °C for 15 min. Lysosomal pH was calculated from a pH standard curve generated using standard 105 mM KCl pH solutions and 10 μM nigericin. 4) CHMP2B recruitment: T-REx-*Tau40*-HEK293 cells were treated with 500 pg/mL doxycycline for 24 h and then with 10 nM TA-3 for 24 h in the absence of doxycycline. Recruitment of CHMP2B, a marker of lysosomal damage repair, was evaluated by immunofluorescence staining using anti-CHMP2B antibody (Proteintech, Rosemont, IO, USA) and anti-LAMP1 antibody as the primary antibody and then with Alexa 594-anti-rabbit IgG and Alexa488-anti-mouse IgG as the secondary antibody. After DAPI staining, CHMP2B puncta colocalized with LAMP1 were quantified by confocal microscopy.

Changes in lysosome after doxycycline treatment were evaluated by immunofluorescence staining with anti-LAMP1 antibody and Alexa 488-anti-mouse IgG. After additional staining with LysoTracker Red DND-99 (Thermo Fisher), cells were subjected to confocal microscopy, and the number and average size of lysosome were determined using built-in programs of ImageJ.

### iPSC (induced pluripotent stem cell)-derived neuronal cells

Generation of iPSCs from a sporadic AD patient and neuronal cell differentiation from iPSCs were conducted as described 30 with modifications. Peripheral blood mononuclear cells (PBMCs) were collected from a 56-year-old female patient with sporadic AD and a 58-year-old female with normal cognition using Histopaque™-1077 PLUS (Sigma-Aldrich). PBMCs were cultured for 4 days in QBSF-60 medium containing 50 μg/mL L-ascorbic acid, 50 ng/mL stem cell factor, 10 ng/mL IL-3, 2 U/mL erythropoietin and 40 ng/mL IGF-1 (all from Thermo Fisher). PMBCs were then infected with Sendai virus vector expressing four reprogramming factors (OCT3/4, SOX2, cMYC, and KLF4) at a multiplicity of infection of 3~10 and transferred into 6-well dishes coated with iMatrix-511 (Matrixome, Osaka, Japan) in pre-iPSC medium comprising 10% FBS, 1% MEM-NEAA, 2 mM L-glutamine, 1% penicillin-streptomycin, 0.1 mM β-mercaptoethanol, 50 μg/mL L-ascorbic acid, and 4 ng/mL basic fibroblast growth factor in DMEM/F12. Over the next 2~4 day, cells were maintained with half-medium changes of the pre-iPSC medium. On day 4, the medium was changed to complete mTeSR™1 medium (Stem Cell Technologies, Vancouver, Canada) via a half-medium change, followed by full medium changes every other day until iPSC-like colonies were formed. Subculturing and expansion were then conducted until the generated iPSCs became stable for characterization and storage, typically at passage 10. Neural progenitor cells (NPCs) were derived from iPSCs using dual SMAD signaling inhibitors [1μM LDN-193189 (Reagents Direct, Encinitas, CA, USA) and 10 μM SB431542 (Reagents Direct)]. NPC identity was confirmed by immunocytochemistry using antibodies to NESTIN (R&D Systems, Minneapolis, MN, USA), SOX2 (Millipore) and MUSASHI (Millipore). NPCs were differentiated into cortical neurons over 8 weeks in Neurobasal A medium containing 1% B27 supplement minus vitamin A, 2 mM Glutamax (all from Thermo Fisher), 10 ng/mL brain-derived neurotrophic factor, 10 ng/mL glial cell-derived neurotrophic factor and 10 ng/mL neurotrophin-3 (all from PeproTech, Cranbury, NJ, USA) on PLO/laminin-coated dishes. This study was approved by the Institutional Review Board of Samsung Medical Center (IRB No. SMC 2013-03-087-052).

### ELISA

Aβ content in cell lysate or supernatant was determined using ELISA kits (R&D systems DAB142 and DAB140), according to the manufacturer's instruction. For competitive determination to assess possible effect of leucomethylene blue (LMB) or TA-3 on Tau level measured by ELISA, 250 μg of recombinant Tau protein (Sigma-Aldrich) was incubated with 1~100 nM of LMB or TA-3 at 37 ℃ for 3 h. After centrifugation at 20,000 x *g* for 10 min, Tau content in the supernatant was determined by ELISA (Invitrogen).

### Cell viability

After treatment of several types of cells with LMB or TA-3 for 72 h, cell viability was examined using a Chromo-CK kit (Monobio, Seoul, Korea).

### Drug affinity responsive target stability (DARTS) assay

Membrane proteins were extracted from T-REx-*Tau40*-HEK293 cells using the Membrane Protein Extraction Kit (Thermo Fisher), according to the manufacturer's instructions. Each protein sample was diluted to the indicated protein concentration and treated with control, LMB or TauAutac-3 (TA-3), followed by incubation on a rotator at 4 °C for 4 h. Samples were treated with DW or 0.3~0.5 μg/mL pronase (Sigma-Aldrich, St. Louis, MO, USA) and incubated for 10 min at RT on a rotator for immunoblot analysis.

### Statistical analysis

All the data are presented as the mean ± SEM. Statistical analysis was performed using GraphPad Prism 9.1.0 software. Two-tailed Student's *t*-test was employed to compare values between two groups. One-way ANOVA with Tukey's *post-hoc* test was used to compare values between multiple groups. Two-way ANOVA with Bonferroni's test was employed to analyze effects of two variables or categories on the outcome. Statistical significance was set at *P* < 0.05.

## Results

### Chemical synthesis of TauAutac (TA)

As autophagic degraders, previously reported lysosomal degradation tags (Autacs) were chosen 31. As a warhead targeting Tau, we have selected methylene blue (MB) that can bind to and block the polymerization of Tau 32. We also synthesized linkers of two to five atoms, and designed five different TAs by combining three parts.

**Figure 1:**
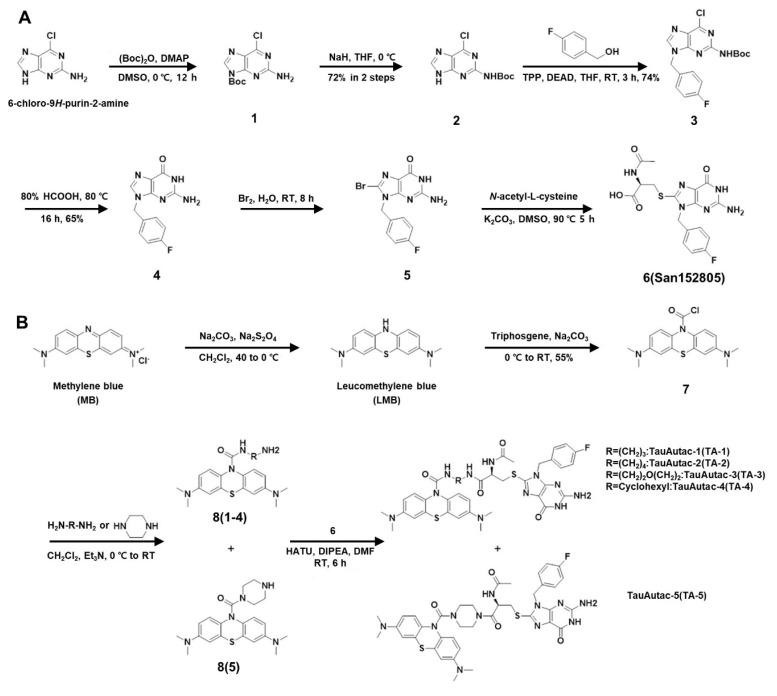
Scheme of the synthesis of TauAutac (TA). **A** Synthesis of the degradation tag. **B** Synthesis of TA using methylene blue (MB), linker and the degradation tag.

To synthesize autophagic degradation tags using to a strategy similar to a reported method 31, six-step synthetic sequence began with Boc protection of 2-amino-6-chloropurine (labelled as **1**), followed by shifting the protection group onto the 2-amino group, yielding compound **2** with 72% yield over two steps **(Figure 1A)**. A Mitsunobu reaction of **2** with 4-fluoro benzyl alcohol gave compound **3** with 74% yield, which was then hydrolyzed with 80% formic acid to produce 2**-**amino-9-(4-fluorobenzyl)-1H-purin-6(9H)-one (labeled as **4**) with 65% yield **(Figure 1A)**. Compound **4** was brominated with 5% bromine water at RT (labelled as **5**), and then coupled with N-acetyl cysteine (NAC) to generate the acid fragment **6** (San152805) with 58% yield in two steps **(Figure 1A)**.

Following completion of tag synthesis, MB warhead was synthesized in two steps. MB (Sigma-Aldrich) was reduced using Na_2_CO_3_/Na_2_S_2_O_4_ to LMB which was reacted *in situ* with triphosgene to obtain stable acid chloride derivate of LMB (labelled as **7**) with 55% yield **(Figure 1B)**. Compound **7** was coupled with several linkers (linear, cyclic or hetero-linear) to obtain various MB-attached linker fragments labelled as **8**(1~5). Finally, amide coupling of **8**(1~5) with acid fragment **6** furnished TA-1~5 **(Figure 1B)**.

### Clearance of Tau by TA

**Figure 2:**
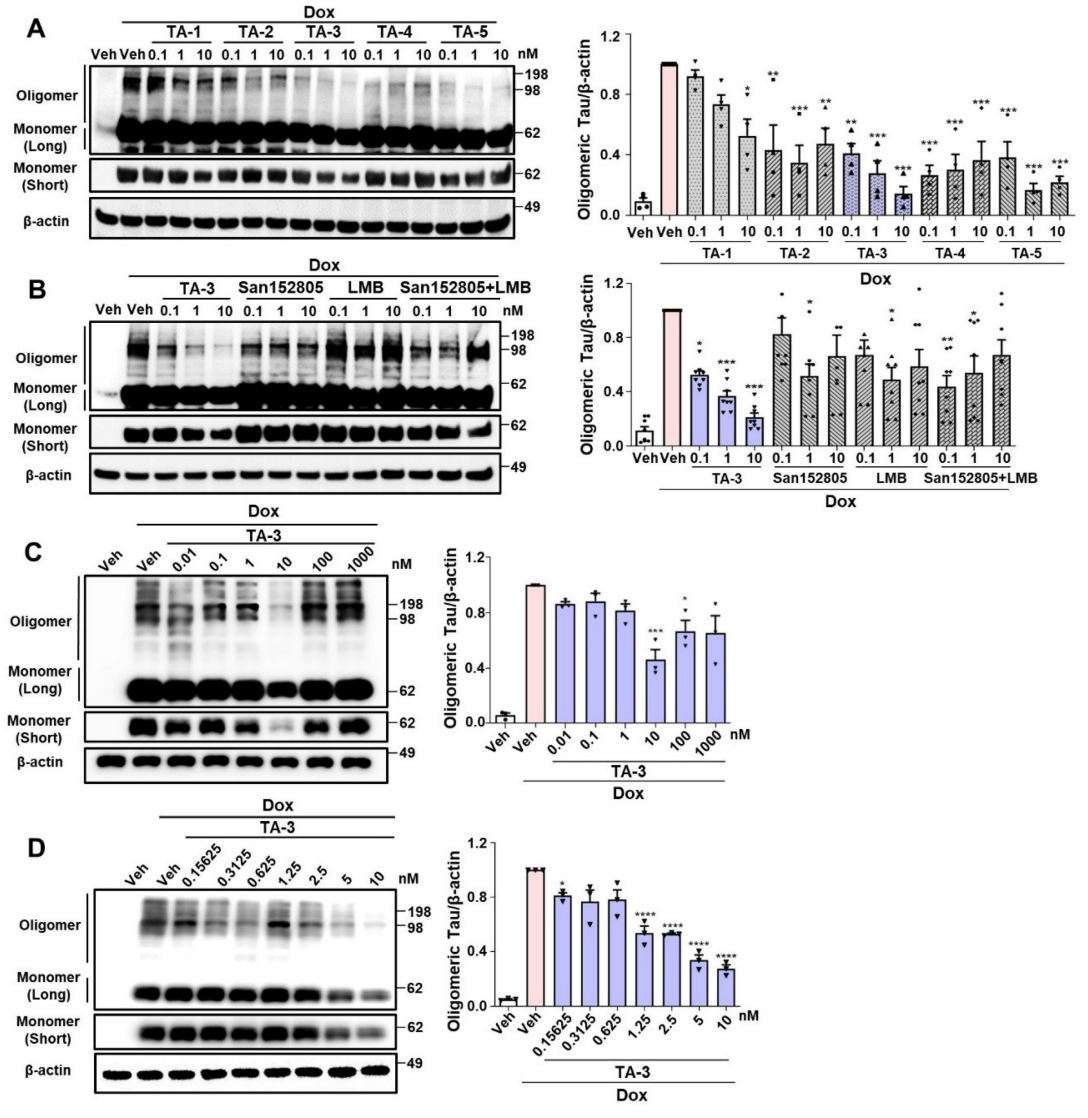
Clearance of Tau by TauAutac (TA). **A** HEK293 cells stably transfected with T-REx*-Tau40* (T-REx-*Tau40*-HEK293 cells) were treated with doxycycline (Dox) for 24 h and then treated with TA for 24 h in the absence of doxycycline. Cell lysates were separated electrophoretically under non-reducing (oligomeric and monomeric Tau) or reducing condition (total Tau), and subjected to immunoblot analysis using anti Tau-5 or β-actin antibody. Densitometric analysis of oligomeric Tau bands was conducted by normalization to β-actin band intensity (right). Representative immunoblots are presented (left). (*n =* 4) **B** After treating T-REx-*Tau40*-HEK293 cells with doxycycline and then with TA-3, degradation tag (San152805), LMB or their combination in the absence of doxycycline, immunoblot analysis was conducted as in (A). Densitometric analysis of oligomeric bands was conducted by normalization to β-actin band intensity (right). Representative immunoblots are presented (left). (*n =* 4) **C**-**D** After treating T-REx-*Tau40*-HEK293 cells with doxycycline and then with 0.01~1,000 nM (C) or 0.15625~10 nM (D) of TA-3 in the absence of doxycycline, immunoblot analysis was conducted as in (A) (each left). Densitometry of oligomer bands was conducted, followed by normalization to β-actin band intensity (each right). (*n =* 4) (*, *P* < 0.05; **, *P* < 0.01; ***, *P* < 0.001; ****, *P* < 0.0001 by one-way ANOVA with Tukey's *post-hoc* test vs. Veh-treated sample after Dox treatment) (Veh, vehicle; Long, long exposure; Short, short exposure).

We studied whether TAs comprising LMB (Tau warhead), a linker and a lysosomal degradation tag could reduce Tau accumulating after treating inducible *Tau*-expressing HEK293 (T-REx-*Tau40*-HEK293) cells 24 with doxycycline for 24 h. All five TAs reduced Tau oligomer accumulation, as identified by electrophoretic mobility on non-reducing gel, to varying degrees. Among them, TA-3 and TA-5 demonstrated the most pronounced effects over 0.1~10 nM concentration range **(Figure 2A)**, exhibiting a high efficacy 31. Additionally, TA-3 significantly reduced accumulation of Tau monomer that is capable of nucleating aggregation depending on the location of intramolecular crosslinking and molecular topography 33 **(Figure 2A and Figure S1A)**. Thus, TA-3 was selected for further experiments. In multiple cells including T-REx-*Tau40*-HEK293 cells, viability was unaffected by various concentrations of TA-3 **(Figure S1B)**, suggesting that TA-3 does not induce cell death at the concentrations employed for *in vitro* experiments and that the decrease of Tau by TA-3 is not associated with cell injury.

The decrease in Tau levels by TA-3 was not due to altered binding of LMB-associated Tau to anti-Tau antibody, since competitive ELISA assay demonstrated no change of measured Tau level after incubation with TA-3 or LMB **(Figure S1C)**. Binding of TA-3 or LMB to Tau was confirmed by a DARTS assay 34, which showed altered susceptibility (or resistance) of Tau to pronase treatment after incubation with LMB or TA-3 likely due to their binding **(Figure S1D**-**E)**, which in consistent with previous results by others 32 and indicates the role of LMB, i.e., the warhead of TA-3 in the binding between TA-3 and Tau. The effect of TA-3 surpassed the combined effect of equimolar concentrations of San152085 (a degradation tag; **6** of Figure 1A) which may induce global autophagic activity through recruitment of K63 and LC3 35, and LMB which can bind to Tau potentially leading to degradation 32 **(Figure 2B)**. These results indicate a synergistic effect of target-specific recognition by a warhead and autophagic degradation of the recognized target by a degradation tag. A dose-response analysis revealed maximal clearance of accumulated Tau oligomer at ~10 nM TA-3 with a half-maximal degradation concentration (DC50) of 1.25 nM **(Figure 2C**-**D)**. At TA-3 concentrations exceeding 100 nM, Tau degradation decreased **(Figure 2C)**, suggesting hook effect that has been observed with MB or Tau Protac 36, 37.

**Figure 3:**
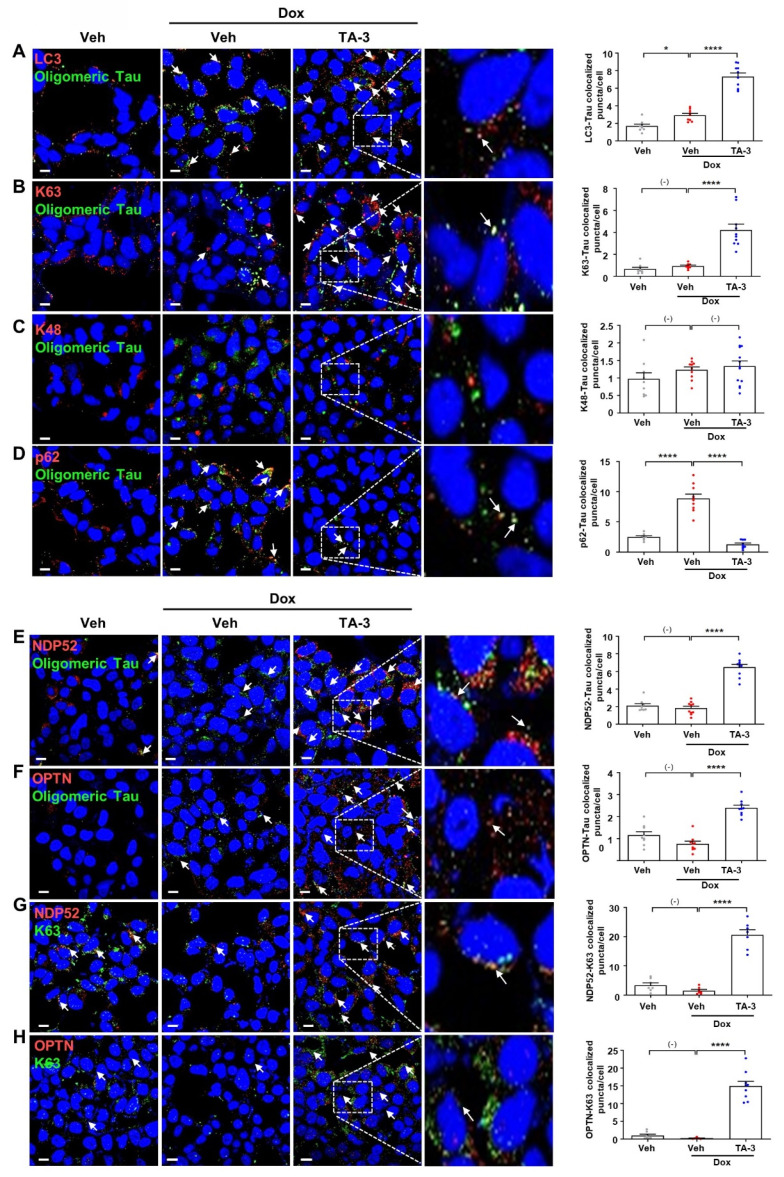
Recruitment of ubiquitin and autophagy receptors by TA-3. **A-F** After treating T-REx-*Tau40*-HEK293 cells with doxycycline (Dox) and subsequently with TA-3 as in Figure 2A, double immunofluorescence staining was conducted by incubating with indicated antibodies (red letters) (A, LC3 antibody; B, anti-K63 antibody; C, anti-K48 antibody; D, anti-p62 antibody; E, anti-NDP52 antibody; F, anti-OPTN antibody) in combination with anti-Tau oligomer antibody (green letters; TOMA-1 or T22), and then with Alexa 488- and Alexa 594-conjugated secondary antibodies (each left). **G-H** Double immunofluorescence staining of cells treated as in A-F was conducted by incubating with antibodies labelled as red letters (G, anti-NDP52 antibody; H, anti-OPTN antibody) and those labelled as green letters (anti-K63 antibody) (each left). Stained sections were analyzed using confocal microscopy after DAPI staining. The numbers of LC3, K63, K48, p62, NDP52 or OPTN puncta colocalized with Tau oligomer in A-F and those of NDP52 or OPTN puncta colocalized with K63 in G-H were quantified (each right). Arrows indicate LC3, K63, K48, p62, NDP52 or OPTN puncta colocalized with Tau oligomer (A-F) and NDP52 or OPTN puncta colocalized with K63 (G-H). (*n =* 10 each) (Scale bar, 10 μm; Rectangles were magnified.) (*, *P* < 0.05; ****, *P* < 0.0001 by one-way ANOVA with Tukey's *post-hoc* test).

We next investigated the mechanism of Tau degradation by TA-3. Reduced Tau accumulation after TA-3 treatment was unrelated to altered Tau expression since *Tau* mRNA expression was not significantly changed by TA-3 **(Figure S2A)**. Clearance of accumulated Tau by TA-3 was diminished by bafilomycin A1 but not by lactacystin **(Figure S2B)**, indicating lysosomal degradation of Tau. Tau clearance by TA-3 was also significantly reduced by *siATG7* **(Figure S2B**-**C)**, supporting TA-3-mediated Tau clearance in a manner dependent on the autophagy-lysosomal system. When we studied recruitment of Atg8 family representing autophagosome elongation/maturation 38, LC3 puncta were formed after TA-3 treatment **(Figure 3A)**, implying autophagic Tau degradation by TA-3. Consistently, a portion of LC3 puncta (~25%) colocalized with Tau oligomer identified using Tau oligomer-specific antibodies 27 **(Figure 3A)**. When we studied possible K63 aggregation which can signal recruitment of autophagy receptors for autophagic degradation 39, K63 puncta were clearly observed after TA-3 treatment, which was partially colocalized with Tau oligomer **(Figure 3B)**. K63 puncta not colocalized with Tau oligomer were also seen. In contrast, K48 aggregation which signals proteasomal degradation 39, was not observed after treatment with TA-3 **(Figure 3C)**, consistent with previous results using other Autac degraders 31. We next studied autophagy receptors that can recognize K63 aggregates and link them to Atg8 molecules for autophagic degradation. Investigation of p62, an important autophagy receptor/adaptor 40, revealed decreased p62 puncta number and fluorescence following TA-3 treatment **(Figure 3D)**, which is in contrast with previous results using other Autac degraders 31 and suggests that p62 is not a crucial adaptor mediating Tau clearance by TA-3. We then studied NDP52 and OPTN, important autophagy receptors for mitophagy or xenophagy 29, 41, 42. Clear NDP52 puncta were observed around Tau oligomer after TA-3 treatment **(Figure 3E)**, suggesting NDP52 recruitment to K63 aggregates. OPTN puncta was also observed after treatment of TA-3 **(Figure 3F)**, suggesting recruitment of OPTN to K63 aggregates as well. Indeed, NDP52 and OPTN puncta colocalized with K63 puncta (~50%) **(Figure 3G-H)**, supporting NDP52 and OPTN recruitment to K63 aggregates. Functional studies employing *NDP52* or *OPTN* knockdown (KD) strategy using *siNDP52* or* siOPTN* revealed that KD of either significantly diminished Tau clearance by TA-3 **(Figure S2D-E)**, emphasizing their pivotal role as dominant autophagy receptors in targeted autophagic Tau clearance by TA-3.

### Amelioration of Tau-induced lysosomal stress *in vitro* by TauAutac

**Figure 4:**
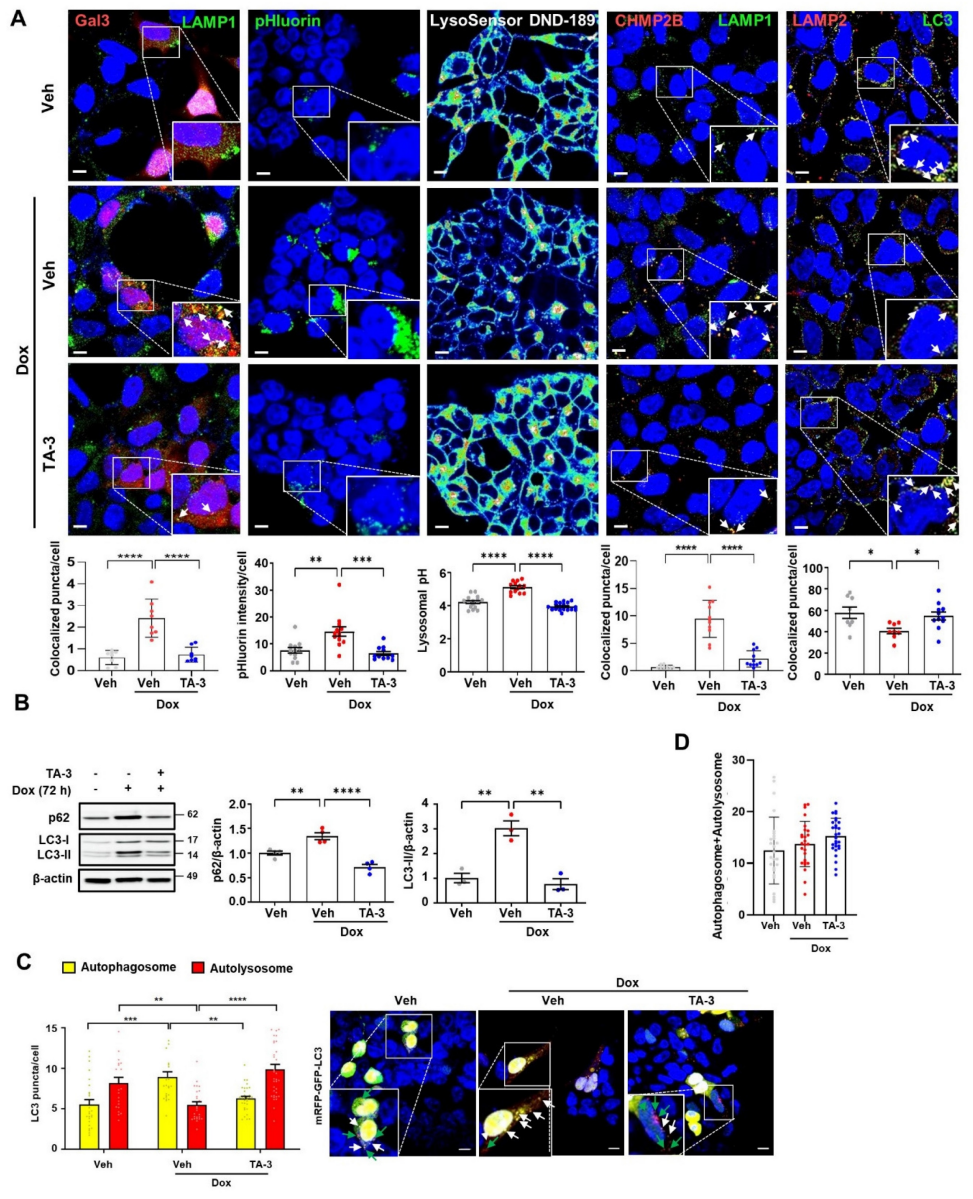
Alleviation of lysosomal stress by TA-3. **A** 1^st^ from the left:* pmCherry-Gal3*-transfected T-REx-*Tau40*-HEK293 cells were treated with doxycycline (Dox) and subsequently with TA-3 as in Figure 2A. After immunofluorescence staining with LAMP1 antibody, the number of galectin-3 puncta colocalized with LAMP1 was quantified following DAPI staining (bottom). 2^nd^ from the left: *CD63-pHluorin*-transfected T-REx-*Tau40*-HEK293 cells were treated as above, and pHluorin fluorescence was quantified (bottom). 3^rd^ from the left: After treatment of LysoSensor DND-189-loaded T-REx-*Tau40*-HEK293 as above, confocal microscopy was conducted to calculate lysosomal pH as described in the Methods (bottom). 4^th^ from the left: T-REx-*Tau40*-HEK293 cells treated as above were subjected to immunofluorescence staining using anti-CHMP2B and anti-LAMP1 antibody as the primary antibodies and then with Alexa 594-anti-rabbit IgG and Alexa 488-anti-mouse IgG as the secondary antibodies. The number of CHMP2B puncta colocalized with LAMP1 was quantified by confocal microscopy (bottom). 5^th^ from the left: After treatment of T-REx-*Tau40*-HEK293 cells as above, double immunofluorescence staining was conducted using anti-LC3 and -LAMP2 antibodies as the primary antibodies. Confocal microscopy was conducted to count the number of LC3 puncta colocalized with LAMP2 (LC3^+^LAMP2^+^ puncta) (bottom). Corresponding representative fluorescence images are presented (top of 1^st^~5^th^ from the left). (Arrows indicate galectin-3, CHMP2 or LC3 puncta colocalized with LAMP1 or LAMP2) (*n =* 10 each) **B** After treating T-REx-*Tau40*-HEK293 cells with doxycycline for 72 h, cells were treated with TA-3 for 24 h in the absence of doxycycline. Cell extract was subjected to immunoblot analysis using indicate antibodies, followed by densitometric analysis of p62 (middle) or LC3-II bands and normalization to β-actin band intensities (right). Representative immunoblots are presented (left). (*n =* 4) **C** T-REx-*Tau40*-HEK293 cells transfected with *mRFP-EGFP-LC3* were treated with doxycycline for 72 h cells, and then with TA-3 for 24 h in the absence of doxycycline. The numbers of yellow and red puncta were quantified by confocal microscopy (left). (*n =* 20) Representative fluorescence images are presented (right) (while arrows, autophagosomes; green arrows, autolysosomes) **D** Sum of yellow puncta and red puncta in each cell. (*n =* 20) (Scale bar, 10 μm; Rectangles were magnified.) (*, *P* < 0.05; **, *P* < 0.01; ***, *P* < 0.001 by one-way ANOVA with Tukey's *post-hoc* test for A,B,D and two-way ANOVA with Bonferroni's test for C).

Since lysosomal dysfunction plays a crucial role in the development of AD 9, 43 and Tau is an important inducer of lysosomal stress 10, 44, we next investigated lysosomal stress caused by Tau and the impact of TA-3 on Tau-mediated lysosomal stress. To assess lysosomal stress, we examined galectin-3 puncta, a marker observed after lysosomal membrane damage, reflecting galectin-3 translocation across damaged lysosomal membrane and its contact with β-galactose-containing glycoproteins in the lysosomal lumen 45. In T-REx-*Tau40*-HEK293 cells transfected with *pmCherry-Gal3* and then treated with doxycycline to induce Tau expression, galectin-3 puncta were well visualized and colocalized with LAMP1, a lysosomal marker **(Figure 4A,** 1^st^ column from the left**)**, indicating Tau-mediated lysosomal stress. The elevated numbers of total and LAMP1-colocalized galectin-3 puncta were significantly downregulated by TA-3 **(Figure 4A,** 1^st^ column from the left**)**, suggesting amelioration of Tau-mediated lysosomal stress through TA-3-mediated diminution of Tau accumulation. We examined another marker of lysosomal stress employing a lysosomal *pHluorin* expression system which relies on changes of pHluorin fluorescence according to the lysosomal luminal pH 46. In T-REx-*Tau40*-HEK293 cells transfected with *CD63-pHluorin* and then treated with doxycycline, pHluorin fluorescence was significantly increased, likely due to Tau-induced lysosomal injury causing a rise in lysosomal pH **(Figure 4A,** 2^nd^ column from the left**)**. Consistent with changes of pHluorin fluorescence, fluorescence of LysoSensor DND-189 fluorescing in acidic pH and lysosomal pH determined by LysoSensor DND-189 fluorescence were decreased and increased, respectively, after doxycycline treatment **(Figure 4A,** 3^rd^ column from the left and **Figure S3A)**, suggesting elevated lysosomal pH and lysosomal dysfunction by Tau. TA-3 treatment significantly reduced pHluorin fluorescence after doxycycline treatment, and restored LysoSensor DND-189 fluorescence normalizing lysosomal pH **(Figure 4A,** 2^nd^ and 3^rd^ columns from the left**)**, indicating amelioration of Tau-mediated lysosomal dysfunction by TA-3, likely through reduced Tau accumulation. We additionally studied puncta of CHMP2B which are recruited to the damaged lysosomal membrane for repair as a component of the ESCRT complex 47. CHMP2B puncta were well visualized in T-REx-*Tau40*-HEK293 cells treated with doxycycline and colocalized with LAMP1, a lysosomal marker, likely due to Tau accumulation inducing lysosomal damage and subsequent repair **(Figure 4A,** 4^th^ column from the left**)**. Increased numbers of total and LAMP1-colocalized CHMP2B puncta returned to basal level after TA-3 treatment **(Figure 4A,** 4^th^ column from the left**)**, suggesting resolution of Tau-induced lysosomal damage by TA-3.

When we examined the characteristics of lysosome that can affect detection of colocalized puncta, the number and size of lysosome, stained with anti-LAMP1 antibody or LysoTracker fluorescing largely in a pH-independent manner, were increased after treatment with doxycycline **(Figure S3B)**, which could be due to adaptive changes in response to lysosomal stress or delayed clearance of damaged lysosome. These results, consistent with previous reports 48, suggests the possibility that increased lysosomal number or mass might contribute to the increased numbers of colocalized puncta between Gal3 and LAMP1 or CHMP2 and LAMP1 after doxycycline treatment; however, we found clear evidence of galectin-3 puncta in *pmCherry-Gal3*-transfected cells and CHMP2B puncta in untransfected cells without LAMP1 costaining **(Figure S3C-D)**, suggesting genuine occurrence of lysosomal stress by Tau irrespective of LAMP1 fluorescence. Lysosomal stress observed in T-REx-*Tau40*-HEK293 cells treated with doxycycline was not due to a potential confounding effect of doxycycline but due to the specific effect of Tau, as doxycycline treatment did not induce lysosomal stress in T-REx-*mTRPM7*-HEK293 cells expressing an irrelevant protein **(Figure S4A)**.

We also studied autophagic flux which is likely affected by lysosomal dysfunction. LC3-II accumulation was observed after doxycycline treatment of T-REx-*Tau40*-HEK293 cells **(Figure 4B)**, which might be due to Tau-induced lysosomal dysfunction and diminished LC3-II degradation associated with impaired lysosomal activity. LC3-II accumulation reached a plateau after 72 h of doxycycline treatment **(Figure S5A)**. This observation of the plateau at 72 h of doxycycline treatment allowed calculation of the coefficient of LC3-II degradation based on the asymptotic LC3-II densitometric values (see Calculation of lysosomal dysfunction index and lysosomal degradation efficiency index in Supplementary Methods) under the assumption that LC3 production is not affected by doxycycline. Indeed, LC3-II accumulation by bafilomycin A, a lysosomal inhibitor, was not further increased by doxycycline **(Figure S5B)**, supporting that LC3-II accumulation after doxycycline treatment is not due to enhanced autophagy and that LC3 production is unaffected by doxycycline. Based on these assumptions, the lysosomal dysfunction index_LC3_ in the presence of doxycycline was calculated to be 3.0, and the lysosomal (autophagic) degradation efficiency index_LC3_ was 1/3.0 (33%). Increased LC3-II accumulation caused by lysosomal dysfunction after doxycycline treatment was significantly downregulated by TA-3 **(Figure 4B)**, completely normalizing lysosomal degradation efficiency (lysosomal dysfunction index_LC3_, 0.75 as calculated from the LC3-II densitometry values in Figure 4B; lysosomal degradation efficiency index_LC3_, 1.33), likely due to the reversal of Tau-mediated lysosomal dysfunction. Similar to LC3-II, accumulation of p62, a representative autophagy substrate 38, was observed after doxycycline treatment, which was significantly reduced by TA-3 **(Figure 4B)**, consistent with the resolution of Tau-mediated lysosomal dysfunction and autophagy impairment by TA-3.

We also investigated colocalization between LC3 and LAMP2, a marker of autophagy progression to the lysosomal step 29. Colocalization between LC3 and LAMP2 was reduced after doxycycline treatment, suggesting lowered autophagic activity likely due to Tau-mediated lysosomal dysfunction **(Figure 4A,** 5^th^ column from the left**)**. TA-3 restored the colocalization between LC3 and LAMP2, suggesting TA-3-mediated recovery of autophagic flux hampered by Tau **(Figure 4A,** 5^th^ column from the left**)**. We additionally evaluated autophagic flux by transfecting T-REx-*Tau40*-HEK293 cells with a fluorescent probe (*mRFP-EGFP-LC3*) allowing quantification of autophagosomes and autolysosomes 49. The number of yellow puncta representing autophagosome and that of red puncta representing autolysosome after doxycycline treatment for 72 h were significantly increased and decreased, respectively, suggesting impaired lysosomal steps of autophagy **(Figure 4C)**. TA-3 treatment significantly decreased and increased numbers of yellow and red puncta, respectively **(Figure 4C)**, suggesting TA-3-mediated recovery of lysosomal steps of autophagy hindered by doxycycline-induced Tau. The sum of the numbers of autophagosomes and autolysosomes identified in *mRFP-EGFP-LC3* transfection study was not significantly affected by doxycycline treatment **(Figure 4D)**, supporting that doxycycline is unlikely to affect steps prior to autophagosome formation *in vitro*.

In contrast to *Tau* induction, *mTRPM7* induction by doxycycline treatment of T-REx-*mTRPM7*-HEK293 cells did not affect LC3-II accumulation **(Figure S4B)**, which is in line with the absence of lysosomal stress in the same condition (See Figure S4A).

### Amelioration of AD phenotype by TA-3 *in vivo*

We next studied *in vivo* effect of TA-3 employing 6xTg mice that express a mutant form (P301L) of Tau in addition to a mutant APP [APPswe/Ind/fl (K670N/M671L/V717F/I716V)] and a mutant PS1 (M146L/L286V) because 6xTg mice develop both amyloid plaques and Tau NFTs at younger ages, coupled with accelerated neurological deficits compared to other AD animal models 19, ensuring study of the effect of TA on the clearance of both Tau and Aβ, together with impact of Tau clearance on the associated neurological deficits. A pharmacokinetic study after single intraperitoneal (i.p.) administration of TA-3 at different doses (1, 5 and 25 mg/kg) revealed that TA-3 concentrations in brain interstitial fluid and whole brain at 24 h (C_last,ISF_) were 10.08 ± 0.67 ng/g (12.33 ± 0.82 nM) and 13.38 ± 0.09 ng/g (16.37 ± 0.11 nM), respectively, in mice treated with 5 mg/kg TA-3 with a brain-to-plasma (B/P) ratio of 0.62 ± 0.07 **(Table S1)**. Since these concentrations were similar to the TA-3 concentration showing maximal Tau clearance *in vitro*, we chose the protocol of i.p. administration of 5 mg/kg TA-3 for further studies.

**Figure 5:**
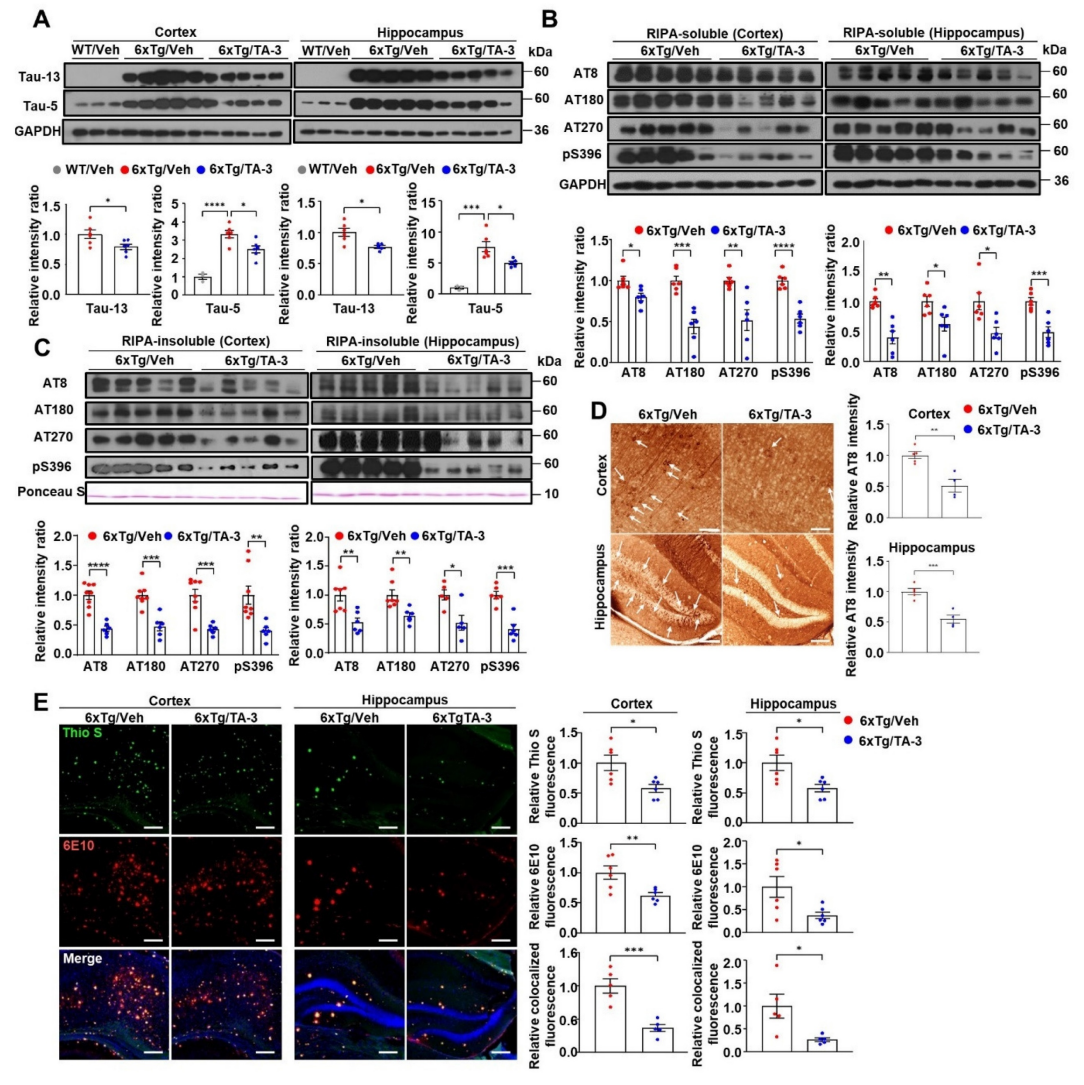
Effects of TA-3 on Tau or Aβ accumulation and amyloid plaques in 6xTg mice. **A** Cortical and hippocampal tissue lysates from 6xTg mice treated with TA-3 or vehicle (Veh) for two months were prepared in RIPA buffer, which were subjected to immunoblot analysis using indicated antibodies recognizing Tau, as described in the Methods (top). Densitometric analysis was conducted by normalizing to GAPDH band intensities (bottom). (*n =* 6~7 per group) **B**-**C** RIPA-soluble (B) and -insoluble fractions (C) prepared from cortical and hippocampal lysate of (A) were subjected to immunoblot analysis using indicated antibodies recognizing phospho-Tau (each top). Densitometric analysis was conducted by normalizing to GAPDH band intensities (each bottom). (*n =* 6~7 per group) **D** Cortical and hippocampal sections of 6xTg mice treated with vehicle (Veh) or TA-3 were subjected to immunohistochemistry using AT8 antibody recognizing phospho-Tau (arrows) (left). (*n =* 4~5 per group) Relative intensity of phospho-Tau staining was quantified using ImageJ software (right). (Scale bar, 200 μm for cortical tissue and 100 μm for hippocampus) **E** Cortical and hippocampal sections from the mice of (A) were subjected to Thioflavin-S (Thio S) staining or immunofluorescence staining using 6E10 antibody (left). Relative fluorescence of Thio S, Aβ or their colocalization was quantified by fluorescence microscopy (right) (*n =* 5 per group). (Scale bar, 100 μm) (Veh, vehicle; WT, wild-type) (*, *P* < 0.05; **, *P* < 0.01; ***, *P* < 0.001, ****, *P* < 0.0001 by two-tailed Student's *t*-test).

We investigated the impact of TA-3 on Tau accumulation in the brain of 6xTg mice. When male 6xTg mice were treated with TA-3 for two months starting at four months of age **(Figure S6A)**, levels of human Tau, recognized by Tau-13 antibody, were significantly downregulated in the cortex and hippocampus compared to vehicle-treated 6xTg mice **(Figure 5A)**. Human Tau recognized by Tau-13 antibody was absent in WT mice **(Figure 5A)**, as expected. Total Tau levels recognized by Tau-5 antibody (specific for 218-225 of human Tau 50) which were significantly increased in the cortex and hippocampus of vehicle-treated 6xTg mice compared to WT mice, were also significantly diminished by TA-3 treatment for two months **(Figure 5A)**. We next studied phospho-Tau, a key hallmark of AD 51. Phospho-Tau was readily detected in RIPA-soluble fraction of the cortex and hippocampus from vehicle-treated 6xTg mice using several antibodies recognizing phosphorylated Ser and/or Thr [AT8, AT180, AT270 and 44-752G(pS396)], which was significantly downregulated by treatment with TA-3 for two months **(Figure 5B)**. Reduction of phospho-Tau was also observed in RIPA-insoluble fraction **(Figure 5C and S7)**, demonstrating effective clearance phospho-Tau by TA-3 in both fractions. In contrast, phospho-Tau, weakly detected in the RIPA-soluble fraction of the cortex from WT mice likely as a physiological process 52, was not changed by TA-3 treatment for two months **(Figure S6B),** reflecting differences between pathological Tau phosphorylation and physiological phosphorylation. Tau in the same fraction of the cortex from WT mice detected by Tau-5 antibody was also not changed by TA-3 treatment for two months **(Figure S6B)**, which is different from the reduced Tau monomer accumulation in doxycycline-treated T-REx-*Tau40*-HEK293 cells by TA-3, likely due to the difference between physiological expression and genetic overexpression of Tau leading to different molecular topography 33. Immunohistochemistry also confirmed that TA-3 treatment significantly reduced accumulation of phospho-Tau stained with AT8 antibody in the cortex and hippocampus **(Figure 5D)**. In these sections stained with AT8 antibody, most of the phospho-Tau^+^ cells were considered to be neuronal cells, based on the morphology such as protrusion indicating axonal projection and contour of cell body, rather than microglia characterized by ramification **(Figure 5D)**. Regarding amyloid, a key characteristic of AD 2, Thioflavin-S staining revealed a significantly reduction in amyloid plaques in the cortex and hippocampus of TA-3-treated 6xTg group compared to vehicle-treated group **(Figure 5E)**. Level of Aβ, a major constituent of amyloid plaques, detected by immunofluorescence staining with 6E10 antibody was also significantly reduced in the cortex and hippocampus of TA-3-treated 6xTg mice **(Figure 5E)**, likely due to amelioration of Tau-mediated lysosomal dysfunction and restoration of autophagic activity by TA-3. Moreover, Thioflavin-S staining colocalized with Aβ was significantly downregulated by TA-3 administration **(Figure 5E)**, suggesting that TA-3 could reduce Aβ and amyloid plaque deposition, likely through restoration of autophagy *in vivo*.

**Figure 6:**
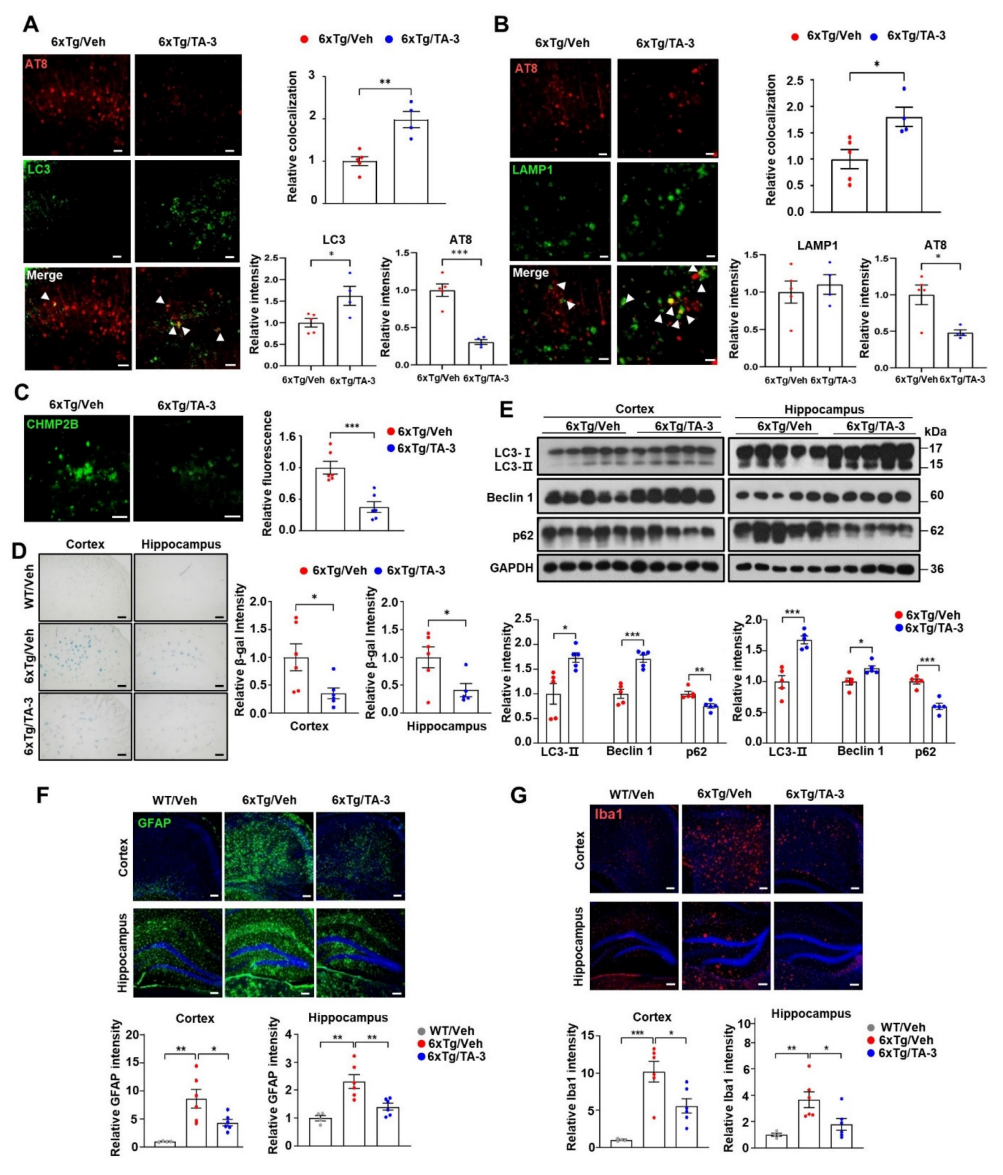
Effects of TA-3 on autophagy, lysosomal stress and neuroinflammation in 6xTg mice.** A**-**B** Brain sections from 6xTg mice treated with vehicle (Veh) or TA-3 for two months were subjected to double immunofluorescence staining using anti-phospho-Tau (AT8) with anti-LC3 (A) or -LAMP1 antibody (B) (each left). Relative each fluorescence and colocalized fluorescence (arrow heads) were quantified by fluorescence microscopy (each right). (*n =* 4~5 per group) **C** Brain sections of (A) were subjected to immunofluorescence staining with anti-CHMP2B antibody (left). Relative fluorescence was determined by fluorescence microscopy (right). (*n =* 4~5 per group) (Scale bar, 100 μm) **D** SA-β-gal staining of cortical and hippocampal tissues from vehicle- or TA-3-treated 6xTg mice was conducted as described in the Methods (left). Relative staining intensity was quantified using ImageJ software (right). (*n =* 6 per group) **E** Cortical and hippocampal tissues lysates from 6xTg mice treated with vehicle or TA-3 were subjected to immunoblot analysis using anti-LC3 and other indicated antibodies (top). Densitometric analysis was performed by normalizing to GAPDH band intensity to calculate the ratio of relative band density in tissues from TA-3-treated mice to that in vehicle-treated tissue (bottom). **F**-**G** Cortical and hippocampal sections from mice of (A) were subjected to immunofluorescence staining using anti-GFAP (F) or -Iba1 antibody (G). After DAPI counterstaining, fluorescence microscopy was conducted to determine relative fluorescence intensity (bottom). Representative fluorescence images are presented (top). (*n =* 4~6 per group) (Scale bar, 100 μm) (*, *P* < 0.05; **, *P* < 0.01; ***, *P* < 0.001 by two-tailed Student's *t*-test).

We next investigated whether Tau degradation by TA-3 occurs through enhanced autophagic activity *in vivo*. Colocalization between phospho-Tau detected by AT8 and LC3 was increased by TA-3 treatment for two months despite the decrease of phospho-Tau **(Figure 6A)**, suggesting autophagic degradation of (phospho-)Tau. Furthermore, colocalization between phospho-Tau and LAMP1, a lysosomal marker, increased with TA-3 treatment despite the decrease of phospho-Tau **(Figure 6B)**, suggesting that accelerated Tau degradation by autophagy proceeds to the lysosomal steps of autophagy. When we examined whether Tau-induced lysosomal stress *in vivo* is changed by TA-3, the number of CHMP2B puncta indicative of lysosomal injury and repair was diminished following TA-3 treatment **(Figure 6C)**, likely due to reduction of Tau attenuating lysosomal stress. Similarly, SA-β-gal activity, another marker of lysosomal dysfunction, which was observed in the cortex and hippocampus of 6xTg mice, was significantly reduced by TA-3 treatment **(Figure 6D)**, supporting amelioration of Tau-mediated lysosomal stress by TA-3. Evidence of lysosomal stress such as SA-β-gal staining or CHMP2B puncta was already observed in the cortex and hippocampus of four-month-old 6xTg mice prior to TA-3 administration, together with amyloid deposition evidenced by Thioflavin-S staining **(Figure S6C-D)**, which indicates that TA-3 treatment started after onset of lysosomal stress, likely mediated by Tau or Aβ.

We also investigated possible changes of autophagic activity *in vivo* by TA-3 since ameliorated lysosomal dysfunction by TA-3 would impact autophagic flux. Immunoblot analysis demonstrated that p62 accumulation, a marker of impaired autophagic flux 38, was significantly reduced in the cortex and hippocampus of TA-3-treated 6xTg mice compared to vehicle-treated 6xTg mice **(Figure 6E)**, suggesting restoration of impaired autophagic activity and amelioration of lysosomal dysfunction. LC3-II accumulation was significantly increased in the cortex and hippocampus of 6xTg mice treated with TA-3 compared to mice treated with vehicle **(Figure 6E)**. This result is distinct from *in vitro* results, and might suggest increased autophagic flux not only at the lysosomal steps but also at the proximal steps of autophagy after prolonged TA-3 treatment *in vivo*. We therefore studied Beclin 1 that acts at the early step of autophagy and can interact with Tau, inducing its sequestration in NFTs 53. Indeed, Beclin 1 levels were significantly elevated in the cortex and hippocampus after TA-3 treatment **(Figure 6E)**, which might be due to liberation of sequestered Beclin 1 from Tau^+^ NFTs or protection of Beclin 1 from Tau-mediated degradation reported in previous studies 53, 54. This increase, which could be due to chronic alteration of the autophagic environment not observed in short-term *in vitro* experiments, might explain the elevated LC3-II levels through activation of the early steps of autophagy, despite amelioration of lysosomal dysfunction by TA-3. Activation of adaptive changes after chronic lysosomal impairment *in vivo* 55, 56 might also contribute to the increased Beclin 1 and LC3-II. Likely due to TA-3-mediated downregulation of Tau or Aβ that can induce inflammasome activation 57, neuroinflammation evidenced by increased numbers of GFAP^+^ astrocytes and Iba1^+^ microglial cells, key players in neuroinflammation of AD 58, 59 was significantly diminished by TA-3 treatment **(Figure 6F**-**G)**.

**Figure 7:**
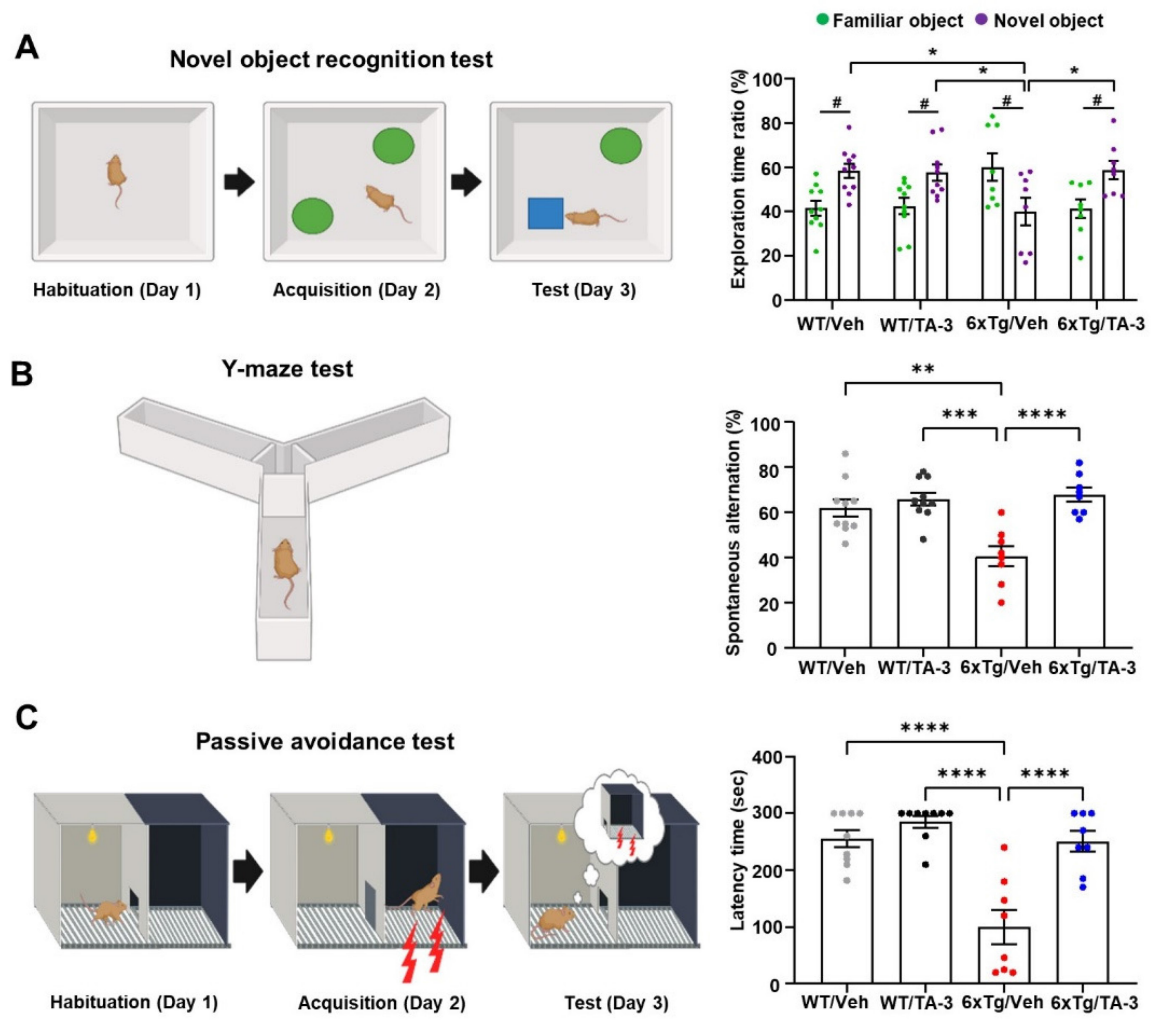
Behavioral test in 6xTg mice treated with TA-3. **A**-**C** Novel object recognition (NOR) (A), Y-maze (B), and passive avoidance (C) tests were performed on wild-type (WT) or 6xTg mice treated with vehicle (Veh) or TA-3 for two months, as described in the Methods. (*n =* 10, 10, 8 and 8 for WT/Veh, WT/TA-3, 6xTg/Veh and 6xTg/TA-3, respectively) (*, *P* < 0.05; **, *P* < 0.01; ***, *P* < 0.001; ****; *P* < 0.0001 between indicated groups; #, *P* < 0.05 vs. familiar object by two-way ANOVA with Bonferroni's test for A and one-way ANOVA with Tukey's *post-hoc* test for B-C).

Having observed reduction of Tau accumulation in the brain of 6xTg mice by TA-3, we next examined whether neurological deficits of 6xTg mice could be ameliorated due to reduced Tau accumulation, using a series of behavioral tests. In the novel object recognition (NOR) test to assess recognition memory, vehicle-treated 6xTg mice spent more time near familiar objects compared to novel objects, whereas WT mice spent more time exploring novel objects **(Figure 7A)**, indicating impaired recognition memory of 6xTg mice. Strikingly, 6xTg mice treated with TA-3 spent more time exploring novel objects than familiar ones, similar to the behavior of WT mice **(Figure 7A)**, demonstrating almost complete recovery of exploration time ratio and recognition memory by TA-3 treatment, likely as a result of reduced Tau and Aβ accumulation in the brain. As control parameters, there was no significant difference in total distance or velocity between groups **(Figure S6E)**. In the Y-maze test, vehicle-treated 6xTg mice displayed significantly reduced spontaneous alternation compared to WT mice, indicating a decreased willingness to explore new environments **(Figure 7B)**. In contrast, TA-3-treated 6xTg mice exhibited significantly improved alternation behavior compared to vehicle-treated 6xTg mice **(Figure 7B)**, suggesting restored exploratory behavior. There was no significant difference in the total number of entry between groups **(Figure S6F)**. In the passive avoidance test (PAT), vehicle-treated 6xTg mice demonstrated significant memory impairment as evidenced by decreased latency time **(Figure 7C)**. In contrast, TA-3-treated 6xTg mice exhibited a significantly increased latency time compared to vehicle-treated 6xTg mice **(Figure 7C)**, indicating beneficial effects of TA-3 against memory impairment in AD.

### Effects of TA-3 on Tau in iPSCs from an AD patient

**Figure 8:**
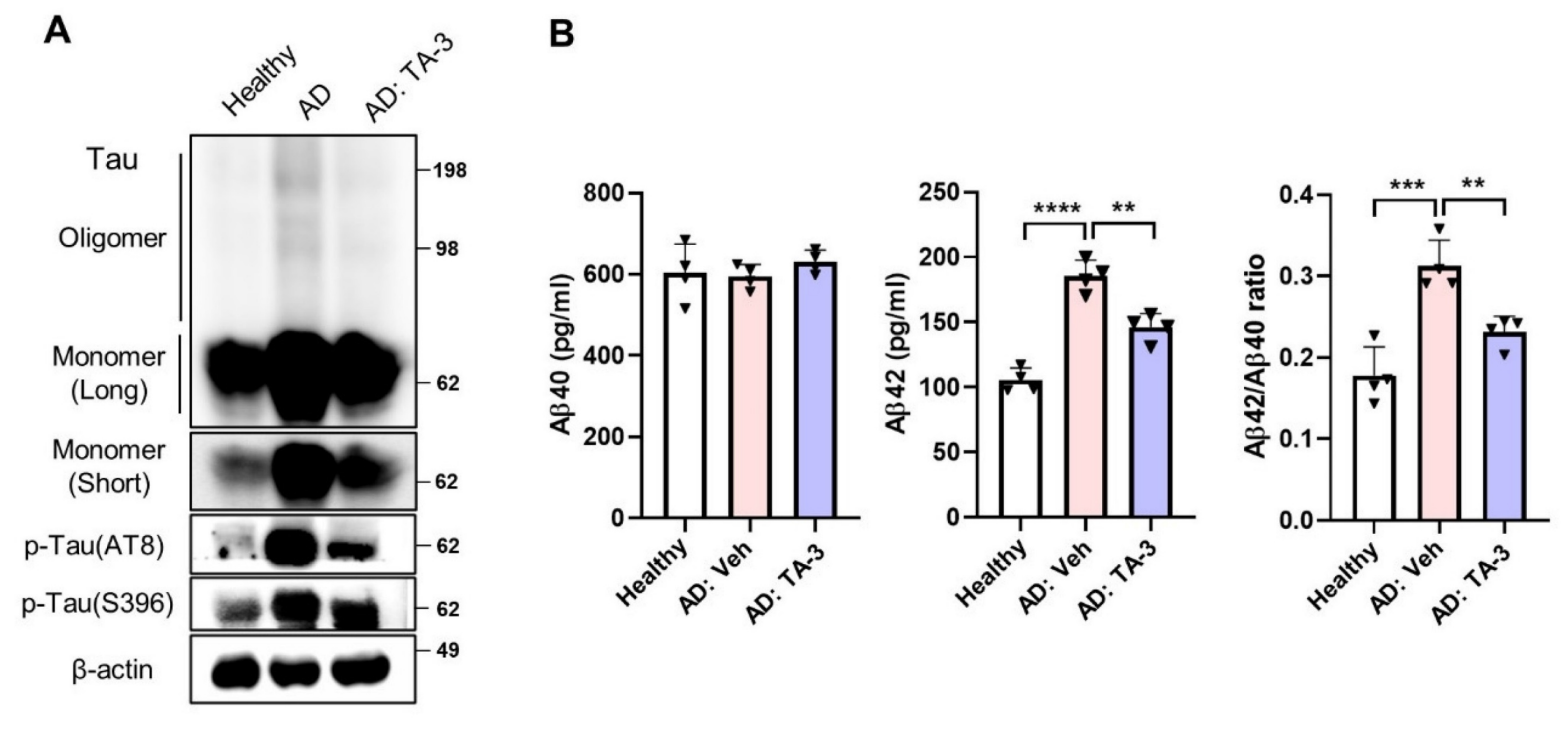
Effect of TA-3 on iPSC-derived neuronal cells from an AD patient. **A** iPSC-derived neuronal cells generated from mononuclear cells of a sporadic AD patient and those of a healthy subject were treated with TA-3 for 24 h. Immunoblot analysis was performed using non-reducing gel and indicated antibodies as in Figures. 2 and 5. **B** ELISA was performed to measure of Aβ40 and Aβ42 levels in the culture supernatant of iPSC-derived neuronal cells of A with or without TA-3 treatment for 24 h. Aβ42/Aβ40 was calculated. (**, *P* < 0.01; ***, *P* < 0.001 by one-way ANOVA with Tukey's post-hoc test) (Veh, vehicle; Long, long exposure; Short, short exposure).

Finally, to ascertain the potential clinical relevance of these findings to human AD, we utilized iPSC-derived neuronal cells generated from mononuclear cells of a sporadic AD patient. Phospho-Tau level was elevated in iPSC-derived neuronal cells from the AD patient compared to those from a healthy subject **(Figure 8A)**. TA-3 treatment significantly reduced phospho-Tau level in iPSC-derived neuronal cells from the AD patient **(Figure 8A)**, suggesting that TA-3 might be able to reduce (phospho-)Tau level in human AD patients. Additionally, Tau oligomer and monomer accumulation which was also elevated in iPSC-derived neuronal cells from the AD patient, was alleviated by TA-3 treatment **(Figure 8A)**. Similarly, Aβ42 level and Aβ42/Aβ40 ratio which were increased in the culture supernatant of iPSC-derived neuronal cells from the AD patient compared to those from a healthy subject, were significantly lowered by TA-3 treatment **(Figure 8B)**, suggesting potential relevance of the therapeutic effects of TA-3 observed* in vitro* and *in vivo* to human AD.

These results collectively suggest that TA-3 could ameliorate neurological deficits of 6xTg mice, likely through reduction of lysosomal stress and autophagy impairment via target-specific Tau clearance. The observed reduction in Aβ accumulation likely due to reduced lysosomal stress or restored autophagic activity, along with diminished neuroinflammation attributable to reduced Tau or Aβ accumulation, might further contribute to the ameliorated neurological deficits in 6xTg mice. Moreover, TA-3 treatment demonstrated the ability to reduce (phospho-)Tau in iPSC-derived neuronal cells of an AD patient, highlighting its potential as a Tau-specific degrader for therapeutic intervention in human AD.

## Discussion

Based on the findings that aggregate or amyloid proteins such as Tau or Aβ are appropriate targets of autophagy/lysosomal degradation, numerous attempts have been conducted to treat AD animal models by enhancing autophagic activity. Most of such efforts have employed strategies aiming at enhancing global autophagy using rapamycin, trehalose or berberine 60, 61, 62. Recently, specific autophagic degradation of Tau has been reported, leading to reduced Tau accumulation in the brain 17. In such a study, a Tau mutant mouse model was employed which is a model of Tauopathy rather than an AD animal model. Furthermore, recovery of neurological deficits or lysosomal dysfunction by Tau-specific autophagic degrader has not been studied. In this investigation, we observed significant amelioration of neurological abnormalities together with markedly reduced accumulation Tau and Aβ using a specific autophagy/lysosomal Tau degrader. Our strategy could be facilitated by employing a mouse model of AD with combined Tau and Aβ mutations which is characterized by accelerated neurological deficits in parallel with Tau accumulation compared to other animal models of AD 19.

The warhead of TA-3 is based on MB that has been employed in clinical trials against AD. As linkers, cyclic linkers with primary or secondary amide linkage were designed, allowing analysis of structure-activity relationship of TAs. TA-1~5 feature linear linkers of 3 to 5 atoms, and TA-3, which incorporates a heteroatomic polyethylene glycol linker, turned out to be the most efficient Tau-specific degrader. While all TA-1~5 reduced Tau accumulation to some extent, TA-3 exhibited remarkably efficient clearance of Tau oligomer and monomer, showing the most dramatic effect within the 0.1~10 nM concentration range. At concentrations above 10 nM, Tau clearance effect was diminished, likely due to hook effect caused by saturation of binding sites on targets or degradation machinery, formation of unproductive binary complex and inhibition of productive ternary complex formation 36, 37. This pronounced efficacy of TA-3 with DC50 of 1.25 nM, which is higher than that of Autac targeting other molecules 31 is likely explained by a productive synergistic interaction between the excellent binding of LMB to Tau 32, the linkers and autophagic degradation tags. The clearance of Tau oligomer or monomer by TA is distinct from natural autophagic clearance. The LMB warhead of TA binds directly and efficiently to Tau oligomer or monomer without recourse to autophagy receptors, unlike natural autophagic processes where most protein aggregates, which are often ubiquitinated, are recognized by autophagy receptors containing LC3-interacting region such as p62 63. After TA binding to soluble Tau oligomer or monomer, K63 ubiquitination occurs, facilitating expedited recruitment of ubiquitin-binding autophagy receptors and subsequent clearance by autophagic/lysosomal pathway. This mechanism can explain efficient clearance of soluble and insoluble Tau by TA.

In addition to its effectiveness in Tau degradation *in vitro* and *in vivo*, TA-3 also reduced Aβ accumulation, which would contribute to the ameliorated neurological deficits in 6xTg mice. The mechanism behind diminished Aβ accumulation by TA-3 administration remains unclear. Several mechanisms could be postulated. First, accumulation of Tau and Aβ may be interrelated. Aβ neurotoxicity can induce Tau phosphorylation. Conversely, Tau accumulation might underlie Aβ accumulation and neurological abnormalities 64. This aligns with previous studies reporting that *Tau* deletion alone ameliorated neurological deficits in AD mouse models 65. Immunization with Tau has also been shown to ameliorate neurological symptoms and pathological changes in 3xTg-AD mouse model, accompanied by reduction in Tau and Aβ 6. Another possibility is that MB facilitates increased Aβ clearance through enhanced proteasomal or mitochondrial activity 66. MB has also been reported to bind Aβ and inhibit Aβ-amyloid assembly 67, which suggests possible effects of MB on the aggregate-prone molecules other than Tau 68 and might contribute to the MB-induced diminution of Aβ accumulation observed in this investigation. However, the most plausible explanation is that TA-3 diminishes Tau-mediated lysosomal dysfunction and consequent autophagy impairment 10, 44, which could enhance autophagic flux and accelerate Aβ clearance. This is supported by findings that Tau-mediated lysosomal stress, evidenced by galectin-3 puncta, increased pHluorin fluorescence associated with altered lysosomal pH and recruitment of CHMP2B, was ameliorated by TA-3. An index of lysosomal dysfunction based on LC3-II accumulation, which was increased by Tau oligomer accumulation, was also reverted by TA-3.

Lysosomal stress or dysfunction might represent an early and fundamental mechanism in AD 9, potentially associated with aging 69, 70, a primary risk factor in sporadic AD 71. On the other hand, early accumulation of Tau or Aβ due to mutations or genetic predisposition might impose lysosomal stress as an initiating event in early-onset AD. A synthesis of these observations suggests that lysosomal dysfunction due to aging or mutation/genetic predisposition induces initial Tau or Aβ accumulation, which, in turn, exacerbates lysosomal stress and dysfunction inducing further accumulation of Tau or Aβ, via a feed-forward mechanism. This cascade would eventually lead to accumulation of large amount of Tau NFTs or fibrillar Aβ/brain amyloid and neurological manifestation. GWAS studies have identified several lysosomal genes associated with early-onset and sporadic AD 72, emphasizing critical roles of lysosomal events in both types of AD.

We could not detect p62 puncta around Tau following TA-3 treatment, contrasting with p62 puncta observed with Autacs targeting other molecules 31. On the other hand, p62 was decreased by TA-3, suggesting enhancement of autophagic activity by TA-3 and degradation of p62 as an autophagy substrate. Notably, instead of p62 puncta, we observed puncta of NDP52 and OPTN-well-known autophagy receptors binding to K63 in several types of selective autophagy such as mitophagy or xenophagy 29, 42 around Tau oligomer. These results suggest that detailed target clearance mechanism of Autac could differ depending on target molecules, proposing a unique model of autophagic clearance tailored to Tau.

Following *in vivo* administration, TA-3 resulted in increased Beclin 1 or LC3-II and decreased p62 in the cortex and hippocampus of 6xTg mice. Increased Beclin 1 in the brain of TA-3-treated mice might result from the reversal of Beclin 1 sequestration or degradation which has been observed in AD brains 53. Increased Beclin 1 might accelerate the early steps of autophagy, potentially leading to elevated LC3-II level, even in the presence of ameliorated lysosomal dysfunction after treatment with TA-3, similar to a previous result 73. In contrast, *in vitro* treatment with TA-3 normalized elevated LC3-II level due to Tau accumulation, suggesting that TA-3 relieves lysosomal stress by removing Tau. This discrepancy between *in vivo* and *in vitro* findings might be attributable to chronic lysosomal stress and prolonged autophagy blockade by Tau oligomer *in vivo*, leading to secondary effects such as Beclin 1 sequestration in Tau NFTs 53, 54. These findings suggest that efficacy of TA-3 *in vivo* might involve not only direct Tau removal but also alleviation of downstream effects associated with chronic lysosomal stress, providing a comprehensive understanding of its therapeutic potential.

Importantly, we observed increased Tau, phospho-Tau and Aβ42 in iPSC-derived neuronal cells from a sporadic AD patient, all of which were attenuated by TA-3. These results suggest the potential relevance of the therapeutic effects of TA-3 observed in experimental animals of AD to human AD, although extensive research will be necessary for the development of actual therapeutic agents based on these findings.

## Conclusion

We conclude that Tau-specific autophagic degraders could enhance clearance of Tau *in vitro* and *in vivo*, along with amelioration of neurological deficits, lysosomal stress and Aβ accumulation. Beneficial effects of Tau-specific autophagic degraders could be extended to iPSC-derived neuronal cells from a sporadic AD patient, suggesting potential therapeutic value of selective autophagic degradation of Tau for treatment of human AD.

## Supplementary Material

Supplementary methods and figures.

## Abbreviations

AD: Alzheimer's disease
BafA: Bafilomycin A1
B/P: brain-to-plasma
DOX: doxycycline
DW: distilled water
ECL: enhanced chemiluminescence
i.p.: intraperitoneally
iPSC: induced pluripotent stem cell
KD: knockdown
Lacta: lactacystin
LMB: leucomethylene blue
MB: methylene blue
NAC: N-acetyl cysteine
NFT: neurofibrillary tangle
NOR: novel object recognition
NPC: neural progenitor cell
PBMC: peripheral blood mononuclear cell
PHF: paired helical filament
RT: room temperature
TA: TauAutac
Tg: transgenic
Thio S: Thioflavin-S
Veh: vehicle
WT: wild-type

## References

[1] Lopez JAS, González HM, Léger GC (2019). Alzheimer's disease. Handb Clin Neurol.

[2] Selkoe DJ, Hardy J (2016). The amyloid hypothesis of Alzheimer's disease at 25 years. EMBO Mol Med.

[3] Shi M, Chu F, Zhu F, Zhu J (2022). Impact of anti-amyloid-β monoclonal antibodies on the pathology and clinical profile of Alzheimer's disease: a focus on Aducanumab and Lecanemab. Fronot Aging Neurosci.

[4] van Dyck CH, Swanson CJ, Aisen P, Bateman RJ, Chen C, Gee M (2023). Lecanemab in Early Alzheimer's Disease. N Eng J Med.

[5] Kametani F, Hasegawa M (2018). Reconsideration of amyloid hypothesis and Tau hypothesis in Alzheimer's disease. Front Neurosci.

[6] Dai C-I, Tung YC, Liu F, Gong C-X, Iqbal K (2017). Tau passive immunization inhibits not only tau but also Aβ pathology. Alzheimers Res Ther.

[7] Chinnathambi S, Malik S, Chandrashekar M (2025). Tau PET probes for Alzheimer's disease detection and their structural characterization. Adv Protein Chem Struct Biol.

[8] Nasrallah IM, Kuo PH, Nordberg A, Bohnen NI, Ponisio MR The Impact of amyloid and Tau PET on Alzheimerdisease diagnostics: AJR expert panel narrative review. AJR Am J Roentgenol. 2025:in press.

[9] Lee JH, Yang DS, Goulbourne CN, Im E, Stavrides P, Pensalfini A (2022). Faulty autolysosome acidification in Alzheimer's disease mouse models induces autophagic build-up of Aβ in neurons, yielding senile plaques. Nature Neurosci.

[10] Jia J, Claude-Taupin A, Gu Y, Choi SW, Peters R, Bissa B (2020). Galectin-3 coordinates a cellular system for lysosomal repair and removal. Dev Cell.

[11] Rubinsztein DC (2006). The roles of intracellular protein-degradation pathways in neurodegeneration. Nature.

[12] Ross CA, Poirier MA (2004). Protein aggregation and neurodegenerative disease. Nat Med.

[13] Lee MJ, Lee JH, Rubinsztein DC (2013). Tau degradation: the ubiquitin-proteasome system versus the autophagy-lysosome system. Prog Neurobiol.

[14] Caballero B, Bourdenx M, Luengo E, Diaz A, Sohn PD, Chen X (2021). Acetylated tau inhibits chaperone-mediated autophagy and promotes tau pathology propagation in mice. Nat Commun.

[15] Iyaswamy A, Wang X, Krishnamoorthi S, Kaliamoorthy V, Sreenivasmurthy SG, Kumar Durairajan SS (2022). Theranostic F-SLOH mitigates Alzheimer's disease pathology involving TFEB and ameliorates cognitive functions in Alzheimer's disease models. Redox Biol.

[16] Kim Y, Choi H, Lee W, Park H, Kam T-I, Hong S-H (2016). Caspase-cleaved tau exhibits rapid memory impairment associated with tau oligomers in a transgenic mouse model. Neurobiol Dis.

[17] Ji CH, Kim HY, Lee MJ, Heo AJ, Park DY, Lim S (2022). The AUTOTAC chemical biology platform for targeted protein degradation via the autophagy-lysosome system. Nat Commun.

[18] Wang W, Zhou Q, Jiang T, Li S, Ye J, Zheng J (2021). A novel small-molecule PROTAC selectively promotes tau clearance to improve cognitive functions in Alzheimer-like models. Theranostics.

[19] Kang S, Kim J, Chang K-A (2021). Spatial memory deficiency early in 6xTg Alzheimer's disease mouse model. Sci Rep.

[20] Brayden DJ, Bzik VA, Lewis AL, Illum L (2012). CriticalSorb™ promotes permeation of flux markers across isolated rat intestinal mucosae and Caco-2 monolayers. Pharm Res.

[21] McGraw T (2016). Polyethylene glycol 3350 in occasional constipation: A one-week, randomized, placebo-controlled, double-blind trial. World J Gastrointest Pathophysiol.

[22] Roche-Molina M, Hardwick B, Sanchez-Ramos C, Sanz-Rosa D, Gewert D, Cruz FM (2020). The pharmaceutical solvent N-methyl-2-pyrollidone (NMP) attenuates inflammation through Krüppel-like factor 2 activation to reduce atherogenesis. Sci Rep.

[23] Charan J, Kantharia ND (2013). How to calculate sample size in animal studies?. J. Pharm Pharmacol.

[24] Lee JH, Shin SK, Jiang Y, Choi WH, Hong C, Kim D-E (2015). Facilitated Tau degradation by USP14 aptamers via enhanced proteasome activity. Sci Rep.

[25] Song C, Choi S, Oh K-B (2020). Suppression of TRPM7 enhances TRAIL-induced apoptosis in triple-negative breast cancer cells. J Cell Physiol.

[26] Castillo-Carranza DL, Sengupta U, Guerrero-Muñoz MJ, Lasagna-Reeves CA, Gerson JE, Singh G (2014). Passive immunization with Tau oligomer monoclonal antibody reverses tauopathy phenotypes without affecting hyperphosphorylated neurofibrillary tangles. J Neurosci.

[27] Lasagna-Reeves CA, Castillo-Carranza DL, Sengupta U, Sarmiento J, Troncoso J, Jackson GR (2012). Identification of oligomers at early stages of tau aggregation in Alzheimer's disease. FASEB J.

[28] Wang JL, Zhao L, Li MQ, Chen W-G, Xu C-J (2020). A sensitive and reversible staining of proteins on blot membranes. Anal Biochem.

[29] Park K, Lim H, Kim J, Hwang H, Lee YS, Bae SH (2022). Essential role of lysosomal Ca2+-mediated TFEB activation in mitophagy and functional adaptation of pancreatic b-cells to metabolic stress. Nat Commun.

[30] Li L, Roh JH, Chang EH, Lee Y, Lee S, Kim M (2018). iPSC modeling of Presenilin1 mutation in Alzheimer's disease with cerebellar ataxia. Exp Neurobiol.

[31] Takahashi D, Moriyama J, Nakamura T, Miki E, Takahashi E, Sato A (2019). AUTACs: cargo-specific degraders using selective autophagy. Mol Cell.

[32] Kiss R, Csizmadia G, Solti K, Keresztes A, Zhu M, Pickhardt M (2018). Structural basis of small molecule targetability of monomeric Tau protein. ASC Chem Neurosci.

[33] Hou Z, Chen D, Ryder BD, Joachimiak LA (2021). Biophysical properties of a tau seed. Sci Rep.

[34] Chang J, Kim Y, Kwon HJ (2016). Advances in identification and validation of protein targets of natural products without chemical modification. Nat Prod Rep.

[35] Ito C, Saito Y, Nozawa T, Fujii S, Sawa T, Inoue H (2013). Endogenous nitrated nucleotide is a key mediator of autophagy and innate defense against bacteria. Mol Cell.

[36] Madan J, Ahuja VK, Dua K, Samajdar S, Ramchandra M, Giri S (2022). PROTACs: Current trends in protein degradation by proteolysis-targeting chimeras. BioDrugs.

[37] Stack C, Jainuddin S, Elipenahli C, Gerges M, Starkova N, Starkov AA (2014). Methylene blue upregulates Nrf2/ARE genes and prevents tau-related neurotoxicity. Hum Mol Genet.

[38] Mizushima N, Komatsu M (2011). Autophagy: renovation of cells and tissues. Cell.

[39] Chen RH, Chen YH, Huang TY (2019). Ubiquitin-mediated regulation of autophagy. J Biomed Sci.

[40] Zaffagnini G, Savova A, Danieli A, Romanov J, Tremel S, Ebner M (2018). p62 filaments capture and present ubiquitinated cargos for autophagy. EMBO J.

[41] Ammanathan V, Mishra P, Chavalmane AK, Muthusamy S, Jadhav V, Siddamadappa C (2020). Restriction of intracellular Salmonella replication by restoring TFEB-mediated xenophagy. Autophagy.

[42] Lazarou M, Sliter DA, Kane LA (2015). The ubiquitin kinase PINK1 recruits autophagy receptors to induce mitophagy. Nature.

[43] Colacurcio DJ, Nixon RA (2016). Disorders of lysosomal acidification-The emerging role of v-ATPase in aging and neurodegenerative disease. Ageing Res Rev.

[44] Midani-Kurçak JS, Dinekov M, Puladi B, Arzberger T, Köhler C (2019). Effect of tau-pathology on charged multivesicular body protein 2b (CHMP2B). Brain Res.

[45] Paz I, Sachse M, Dupont N, Mounier J, Cederfur C, Enninga J (2010). Galectin-3, a marker for vacuole lysis by invasive pathogens. Cell Microbiol.

[46] Ponsford AH, Ryan TA, Raimondi A, Cocucci E, Wycislo SA, Fröhlich F (2020). Live imaging of intra-lysosome pH in cell lines and primary neuronal culture using a novel genetically encoded biosensor. Autophagy.

[47] Skowyra ML, Schlesinger PH, Naismith TV, Hanson PI (2018). Triggered recruitment of ESCRT machinery promotes endolysosomal repair. Science.

[48] Eriksson I, Wäster P, Öllinger K (2020). Restoration of lysosomal function after damage is accompanied by recycling of lysosomal membrane proteins. Cell Death Dis.

[49] Kimura S, Noda T, Yoshimori T (2007). Dissection of the autophagosome maturation process by a novel reporter protein, tandem fluorescent-tagged LC3. Autophagy.

[50] Porzig R, Singer D, Hoffmann R (2007). Epitope mapping of mAbs AT8 and Tau5 directed against hyperphosphorylated regions of the human tau protein. Biochem Biophys Res Com.

[51] Busche MA, Hyman BT (2020). Synergy between amyloid-β and tau in Alzheimer's disease. Nature Neurosci.

[52] Kimur T, Ishiguro K, Hisanaga S-I (2014). Physiological and pathological phosphorylation of tau by Cdk5. Front Mol Neurosci.

[53] Salminen A, Kaarniranta K, Kauppinen A, Ojala J, Haapasalo A, Soininen H (2013). Impaired autophagy and APP processing in Alzheimer's disease: The potential role of Beclin 1 interactome. Prog Neurobiol.

[54] Rohn TT, Wirawan E, Brown RJ, Harris JR, Masliah E, Vandenabeele P (2011). Depletion of Beclin-1 due to proteolytic cleavage by caspases in the Alzheimer's disease brain. Neurobiol Dis.

[55] Bordi M, Berg MJ, Mohan PS, Peterhoff CM, Alldred MJ, Che S (2016). Autophagy flux in CA1 neurons of Alzheimer hippocampus: Increased induction overburdens failing lysosomes to propel neuritic dystrophy. Autophagy.

[56] Settembre C, Ballabio A (2014). Lysosomal adaptation: how the lysosome responds to external cues. Cold Spring Harb Pespect Biol.

[57] Ising C, Venegas C, Zhang S, Scheiblich H, Schmidt SV, Vieira-Saecker A (2019). NLRP3 inflammasome activation drives tau pathology. Nature.

[58] Chun H, Lee CJ (2018). Reactive astrocytes in Alzheimer's disease: A double-edged sword. Neurosci Res.

[59] Venegas C, Kumar S, Franklin BS, Dierkes T, Brinkschulte R, Tejera D (2017). Microglia-derived ASC specks cross-seed amyloid-β in Alzheimer's disease. Nature.

[60] Du J, Liang Y, Xu F, Sun B, Wang Z (2013). Trehalose rescues Alzheimer's disease phenotypes in APP/PS1 transgenic mice. J. Pharm Pharmacol.

[61] Majumder S, Richardson A, Strong R, Oddo S (2011). Inducing autophagy by rapamycin before, but not after, the formation of plaques and tangles ameliorates cognitive deficits. PLOS ONE.

[62] Sun C, Gao X, Sha S, Wang S, Shan Y, Li L (2025). Berberine alleviates Alzheimer's disease by activating autophagy and inhibiting ferroptosis through the JNK-p38MAPK signaling pathway. Int J Pharm.

[63] Johansen T, Lamark T (2020). Selective autophagy: ATG8 family proteins, LIR motifs and cargo receptors. J Mol Biol.

[64] Kam T-I, Park H, Gwon Y, Song S, Kim S-H, Moon SW (2016). FcγRIIb-SHIP2 axis links Aβ to tau pathology by disrupting phosphoinositide metabolism in Alzheimer's disease model. eLife.

[65] Roberson ED, Scearce-Levie K, Palop JJ, Yan F, Cheng IH, Wu T (2007). Reducing endogenous tau ameliorates amyloid beta-induced deficits in an Alzheimer's disease mouse model. Science.

[66] Zakaria A, Hamdi N, Abdel-Kader RM (2016). Methylene blue improves brain mitochondrial ABAD functions and decreases Aβ in a neuroinflammatory Alzheimer's disease mouse model. Mol Neurobiol.

[67] Lee BI, Suh YS, Chung YJ, Yu K, Park CB (2017). Shedding light on Alzheimer's β-amyloidosis: Photosensitized methylene blue inhibits self-assembly of β-amyloid peptides and disintegrates their aggregates. Sci Rep.

[68] Yamashita M, Nonaka T, Arai T, Kametani F, Buchman VL, Ninkina N (2009). Methylene blue and dimebon inhibit aggregation of TDP-43 in cellular models. FEBS Lett.

[69] Geng YQ, Guan JT, Xu XH, Fu YC (2010). Senescence-associated beta-galactosidase activity expression in aging hippocampal neurons. Biochem Biophys Res Com.

[70] Hütter E, Skovbro M, Lener B, Prats C, Rabøl R, Dela F (2007). Oxidative stress and mitochondrial impairment can be separated from lipofuscin accumulation in aged human skeletal muscle. Aging Cell.

[71] Hou Y, Dan X, Babbar M, Wei Y, Hasselbalch SG, Croteau DL (2019). Ageing as a risk factor for neurodegenerative disease. Nat Rev Neurol.

[72] Van Acker ZP, Bretou M, Annaert W (2019). Endo-lysosomal dysregulations and late-onset Alzheimer's disease: impact of genetic risk factors. Mol Neurodegener.

[73] Wang J, Liu B, Xu Y, Yang M, Wang C, Song M (2021). Activation of CREB-mediated autophagy by thioperamide ameliorates β-amyloid pathology and cognition in Alzheimer's disease. Aging Cell.

