# Supplementary material for: Targeted autophagic clearance of Tau protects against Alzheimer's disease through amelioration of Tau-mediated lysosomal stress
Source: Theranostics. 2025 Aug 16;15(17):9240–60. doi: 10.7150/thno.118409 (PMC12439469; doi:10.7150/thno.118409)
Supplement: Supplementary file 1 — Supplementary methods and figures. [file thnov15p9240s1.pdf]

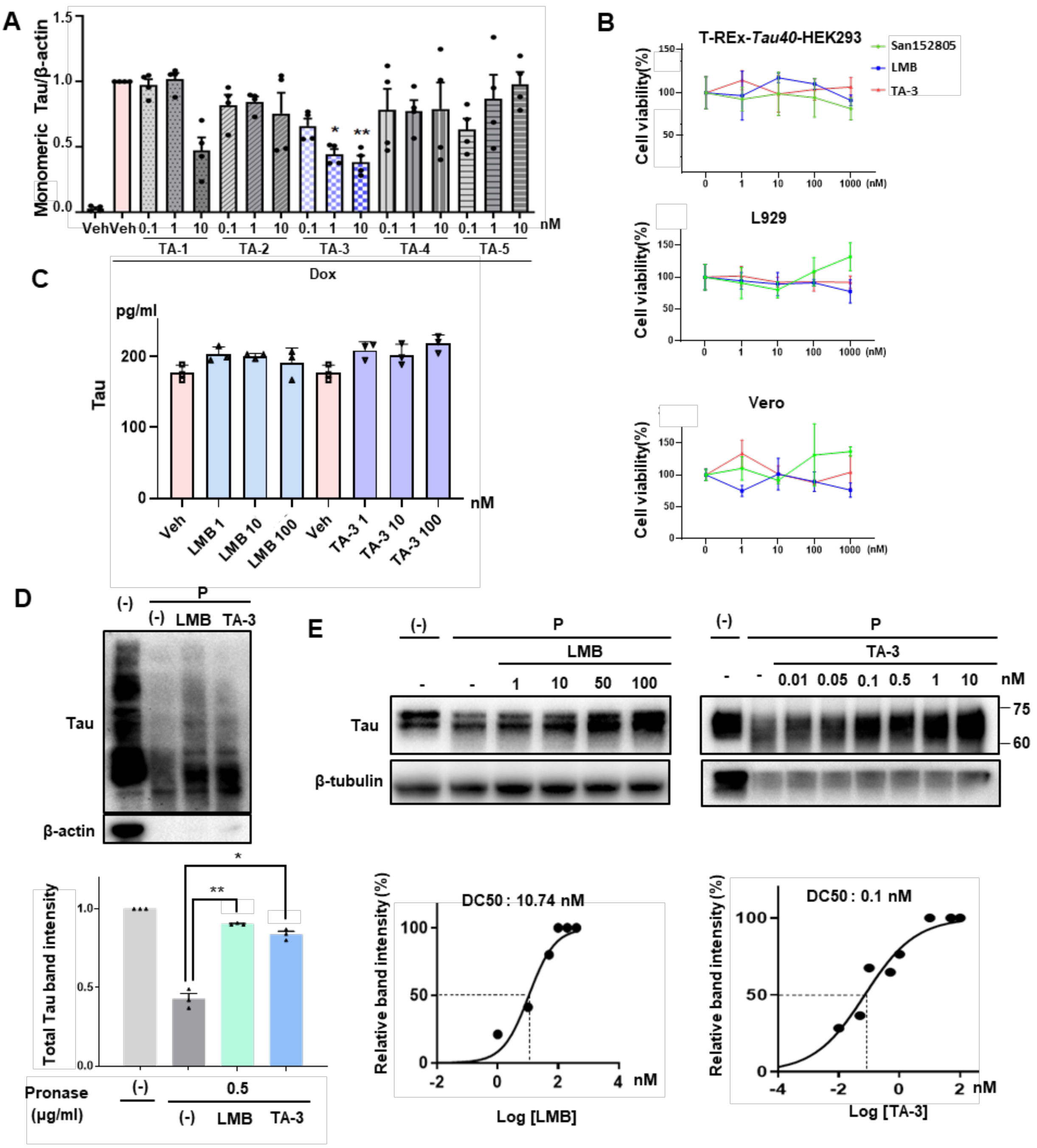

Figure S1 Tau monomer clearance by TAs and Tau binding of TA-3. (A) Densitometric analysis of monomeric Tau bands in the immunoblots of Figure 2A (Short exposure) was conducted by normalization to β-actin band intensity. (n = 4 each) (B) After treating T-REx-*Tau40*-HEK293, L929 and Vero cells with TA-3 of the indicated concentrations for 72 h, cell

viability was evaluated using a Chromo-CK kit. (C) After incubating human Tau protein with LMB or TA-3 of the indicated concentrations and centrifugation, human Tau content in the supernatant was assessed by ELISA as described in the Methods. (D) Binding affinity of LMB and TA-3 to Tau was assessed by drug affinity responsive target stability (DARTS) assay. After treating T-REx-*Tau40*-HEK293 cells with 2 ng/mL of doxycycline for 48 h, cell lysate (1 mg/mL) was incubated with 10 nM of LMB or TA-3 at 4 °C for 4 h, followed by treatment with 0.5 μg/mL pronase for 10 min. After electrophoretic separation, immunoblot analysis using indicated antibodies including Tau-5 antibody (top). Densitometric analysis of total Tau bands was performed by normalization to respective β-actin band intensity (bottom). (E) After the same treatment as in D, cell lysates (1 mg/mL) were incubated with indicated concentrations of LMB (left) or TA-3 (right) at 4 °C for 4 h, followed by treatment with 0.5 μg/mL pronase for 10 min. After electrophoretic separation and immunoblot analysis using Tau-5 antibody (top), densitometric analysis of monomeric Tau bands was performed by normalization to respective control band intensity for calculation of the half-maximal degradation concentration (DC_{50}) (bottom). (*, $P < 0.05$; **, $P < 0.01$ by one-way ANOVA with Tukey's *post-hoc* test)

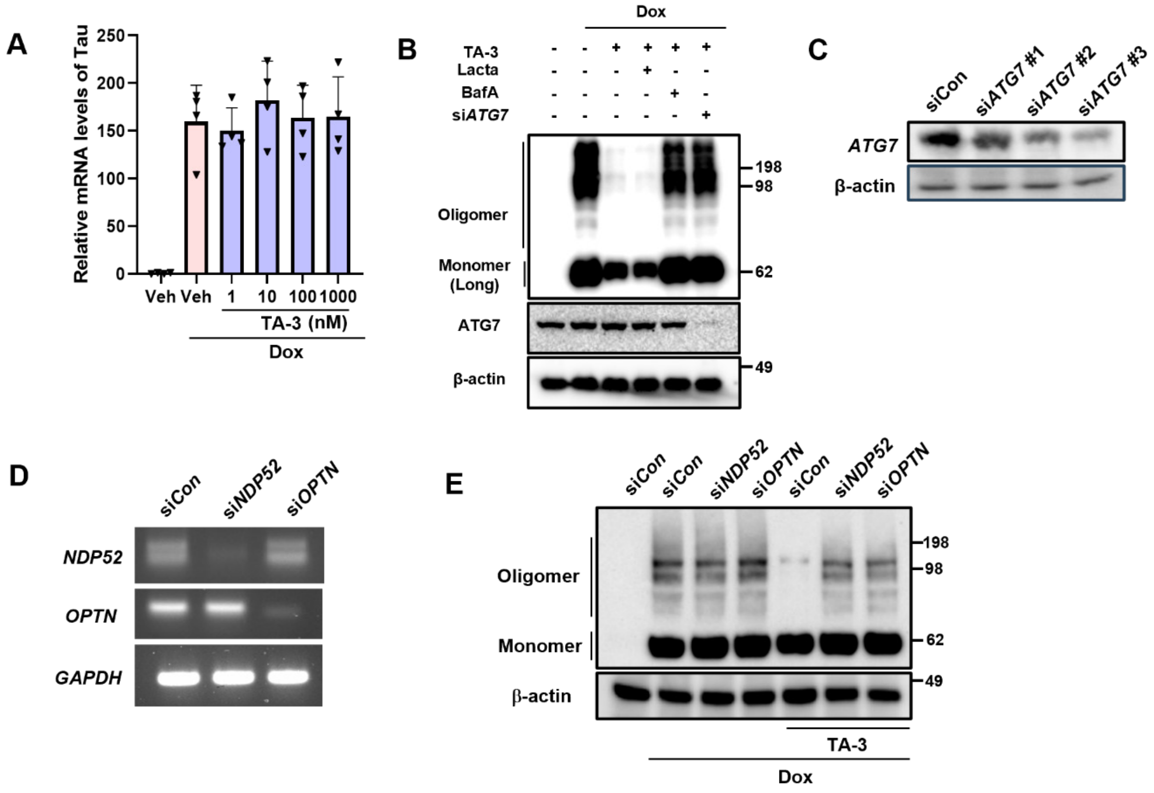

Figure S2 *Tau* mRNA expression and effect of *NDP52* or *OPTN* KD. (A) *Tau* mRNA expression was evaluated by real-time RT-PCR after treating T-REx-*Tau40*-HEK293 cells with of doxycycline in the presence or absence of the indicated concentrations of TA-3 for 24 h. (B) After treating T-REx-*Tau40*-HEK293 cells with doxycycline for 24 h and then with 10 nM of TA-3 in the presence or absence of 5 nM bafilomycin A1 (BafA) or 1 μM lactacystin (Lacta) without doxycycline for another 24 h, immunoblot analysis was conducted using Tau-5 antibody as in Figure 2A. To study effect of si*ATG7*, T-REx-*Tau40*-HEK293 transfected with si*ATG7*#3 were treated with doxycycline and then with TA-3 in the absence of doxycycline. (C) Effect of si*ATG7* on *ATG7* expression in T-REx-*Tau40*-HEK293 cells was evaluated by RT-PCR. (D) After KD of *NDP52* or *OPTN* in T-REx-*Tau40*-HEK293 cells as described in the Methods, expression of *NDP52* and *OPTN* was examined by RT-PCR. (E) T-REx-*Tau40*-HEK293 cells with KD of *OPTN* or *NDP52* were treated with doxycycline (Dox) for 24 h and

then with TA-3 for another 24 h in the absence of doxycycline. Cell extract was subjected to immunoblot analysis using indicated antibodies as in B.

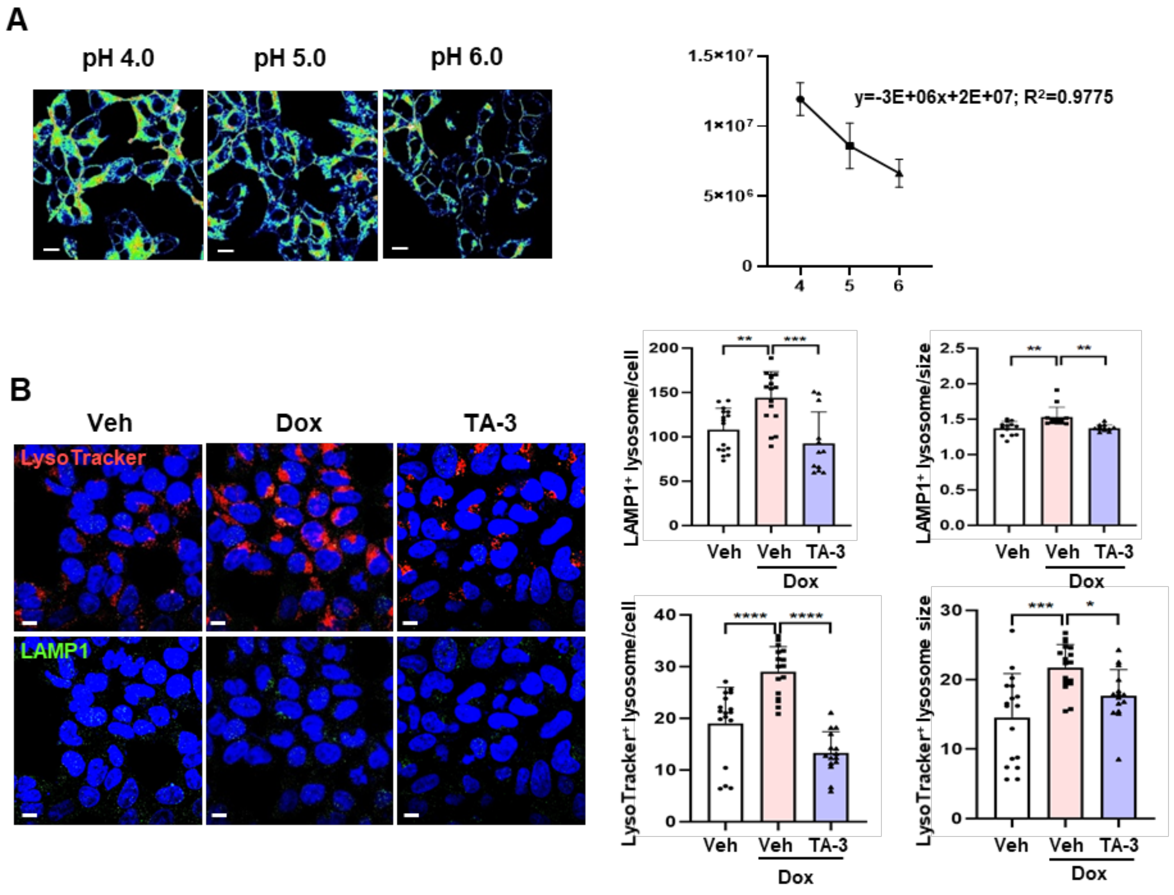

Figure S3 Changes of lysosomal number or size by Tau, and standard curve of lysosomal pH. (A) After treating LysoSensor DND-189-loaded T-REx-*Tau40*-HEK293 cells with standard pH solutions and 10 μM nigericin, LysoSensor DND-189 fluorescence was determined to derive a standard pH curve (right). Representative fluorescence images are presented (left). (Scale bar, 10 μm) (B) After treating T-REx-*Tau40*-HEK293 cells with 500 pg/mL doxycycline and then with TA-3 as in A, cells were incubated with anti-LAMP1 antibody and then with Alexa 488-anti mouse IgG. After further incubation with LysoTracker Red DND-99 and DAPI, cells were subjected to confocal microscopy, and the number and size of LAMP1^{+} or LysoTracker^{+} lysosomes were determined by ImageJ (bottom). Representative fluorescence images are presented (top). (Scale bar, 10 μm)

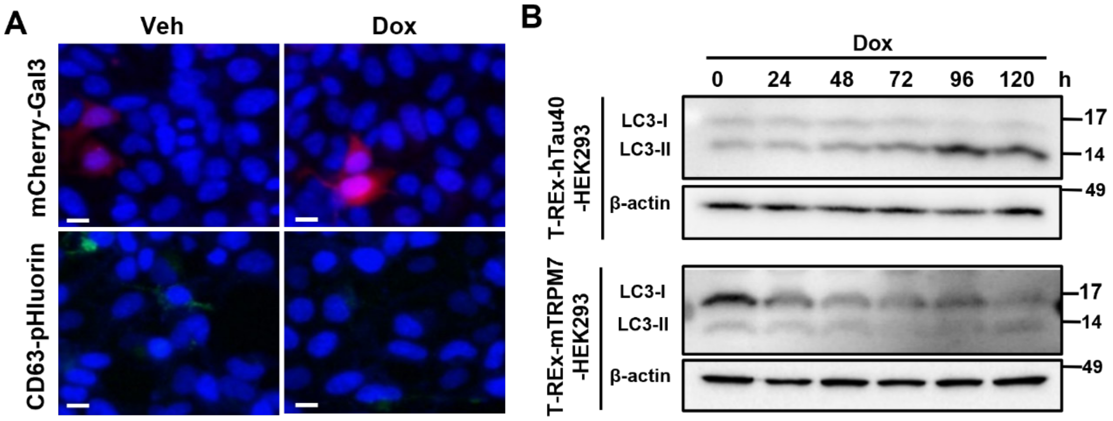

Figure S4 Absence of lysosomal stress and no changes of autophagic flux by doxycycline-induced expression of an irrelevant protein. (A) *pmCherry-Gal3*- (top) or *CD63-pHluorin*-transfected (bottom) T-REx-*mTRPM7*-HEK293 cells were treated with 500 pg/mL doxycycline for 24 h and then with 10 nM TA-3 for 24 h in the absence of doxycycline. After DAPI staining, confocal microscopy was conducted to visualize galectin-3 puncta or pHluorin fluorescence as in Figure 4A. (Scale bar, 10 μm) (B) After treating T-REx-*Tau40*-HEK293 cells and T-REx-*mTRPM7*-HEK293 cells with 500 pg/mL doxycycline for up to 120 h, immunoblot analysis was conducted using anti-LC3 and -β-actin antibodies.

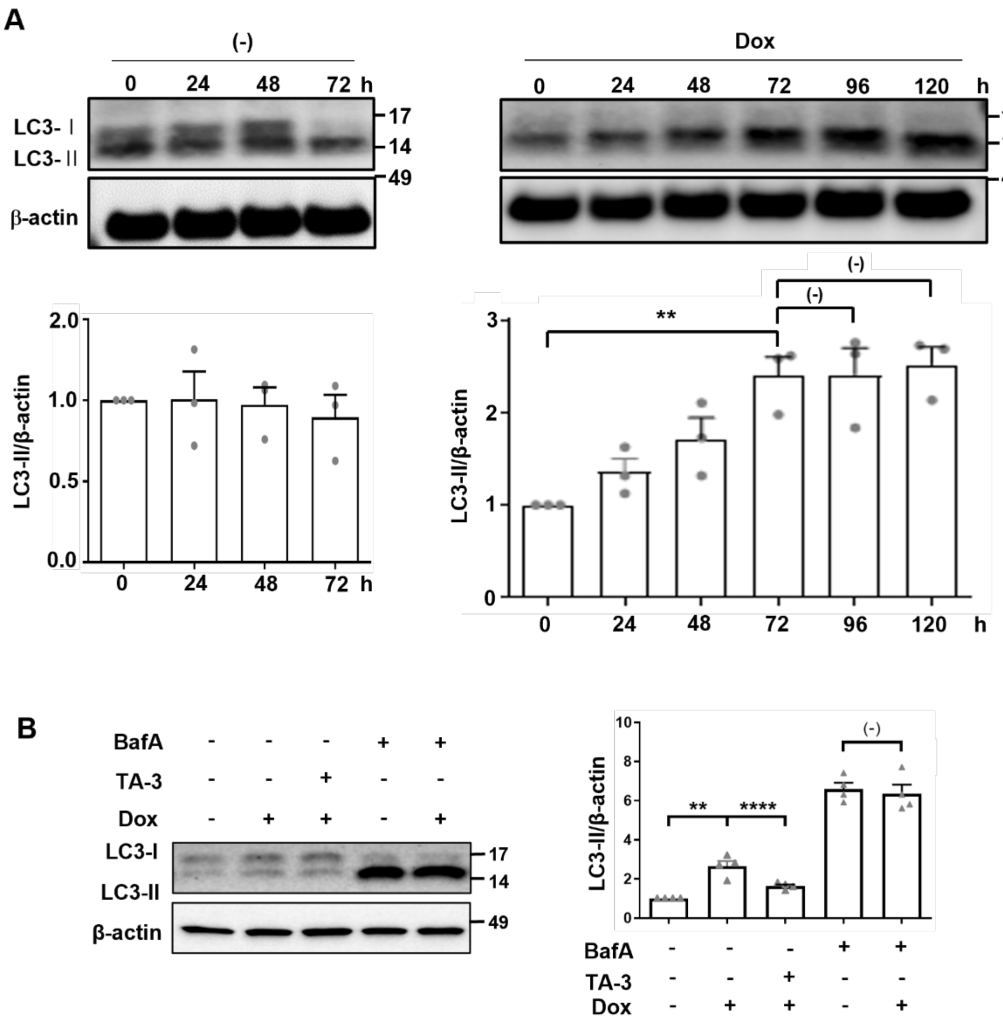

Figure S5 Accumulation of LC3-II by Tau expression. (A) After doxycycline (Dox) treatment of T-REx-*Tau40*-HEK293 for up to 120 h, cell lysate was subjected to immunoblot analysis using anti-LC3 and -β-actin antibodies (top). Densitometry of the LC3-II bands was conducted followed by normalization to the β-actin band density (bottom). (n = 3) (B) Lysates prepared from T-REx-*Tau40*-HEK293 cells treated with 5 nM bafilomycin A1 (BafA) in the presence or absence of Dox for 24 h, were subjected to immunoblot analysis using anti-LC3 and -β-actin antibodies (left). Cells not treated with BafA were employed as controls. Densitometry was conducted, followed by normalization to β-actin band intensities. (**, P < 0.01; ****, P < 0.0001 by one-way ANOVA with Tukey's *post-hoc* test)

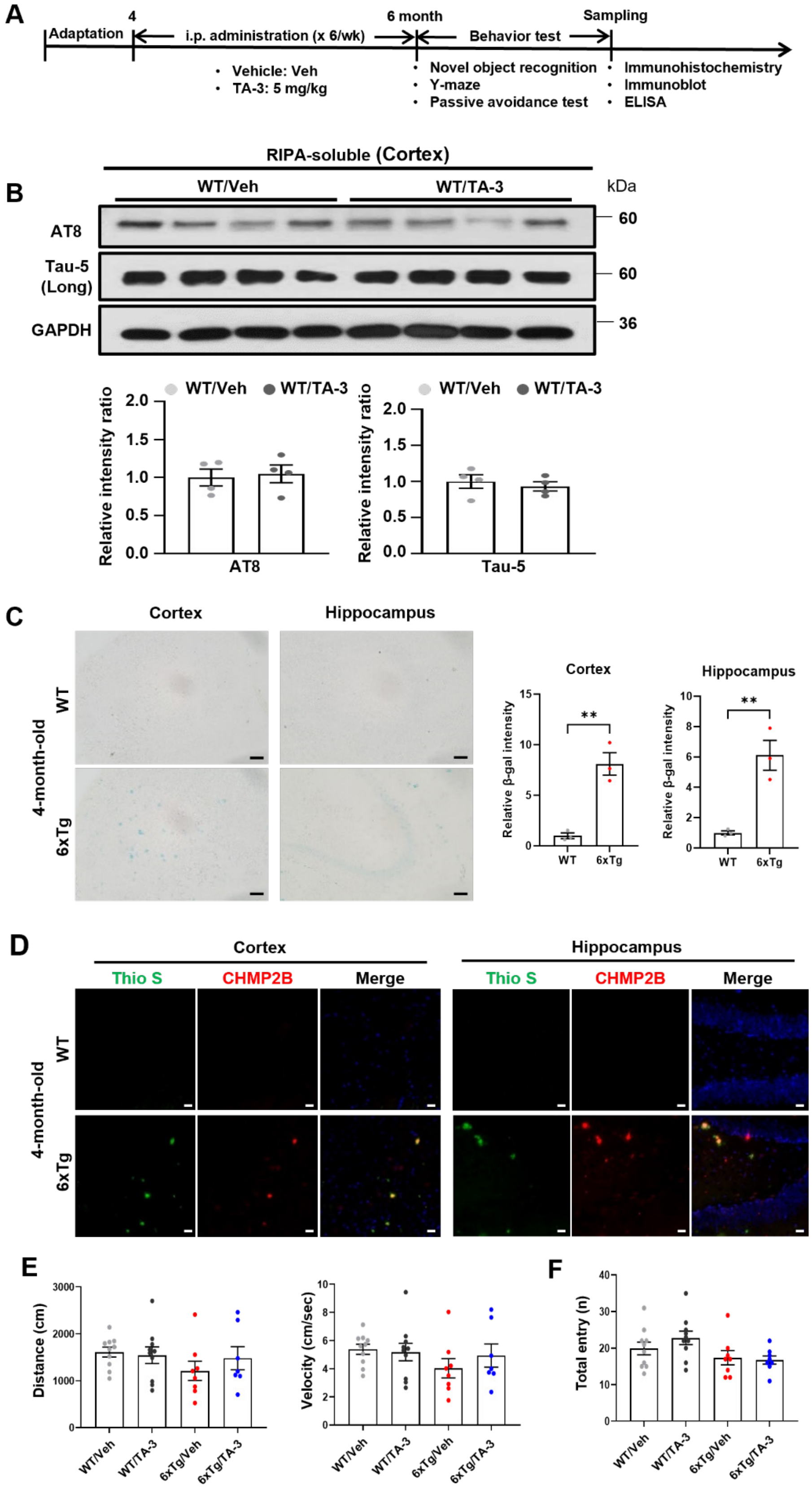
A
Adaptation
4
i.p. administration (x 6/wk)
6 month
Behavior test
Sampling
Vehicle: Veh
TA-3: 5 mg/kg
Novel object recognition
Y-maze
Passive avoidance test
Immunohistochemistry
Immunoblot
ELISA
RIPA-soluble (Cortex)
B
WT/Veh
WT/TA-3
kDa
AT8
Tau-5 (Long)
GAPDH
60
60
36
Relative intensity ratio
AT8
Tau-5
C
Cortex
Hippocampus
4-month-old
WT
6xTg
Relative β-gal intensity
D
Thio S
CHMP2B
Merge
E
Distance (cm)
Velocity (cm/sec)
WT/Veh
WT/TA-3
6xTg/Veh
6xTg/TA-3
F
Total entry (n)

Figure S6 Scheme of *in vivo* treatment and neurological evaluation. (A) Time of *in vivo* administration of TA-3 and evaluation of therapeutic effects. (B) RIPA-soluble fraction of cortical tissue lysate from WT mice treated with TA-3 or vehicle (Veh) for two months was subjected to immunoblot analysis using indicated antibodies recognizing phospho-Tau and total Tau as described in the Methods (top). Densitometric analysis was conducted by normalizing to GAPDH band intensities (bottom). (C) SA-β-gal activity was determined in the cortical and hippocampal sections from 4-month-old 6xTg mice as in Figure 6D (left), followed by quantification using ImageJ (right). (D) The same sections were subjected to Thioflavin-S staining and immunofluorescence staining using anti-CHMP2B antibody to visualize amyloid accumulation and CHMP2B puncta, respectively. (E-F) Parameters of novel object recognition (NOR) test (E) and Y-maze test (F). (*n* = 10, 10, 8 and 8 for WT/Veh, WT/TA-3, 6xTg/Veh and 6xTg/TA-3, respectively) (Scale bar, 100 μM) (See Methods for a detailed description of test methods)

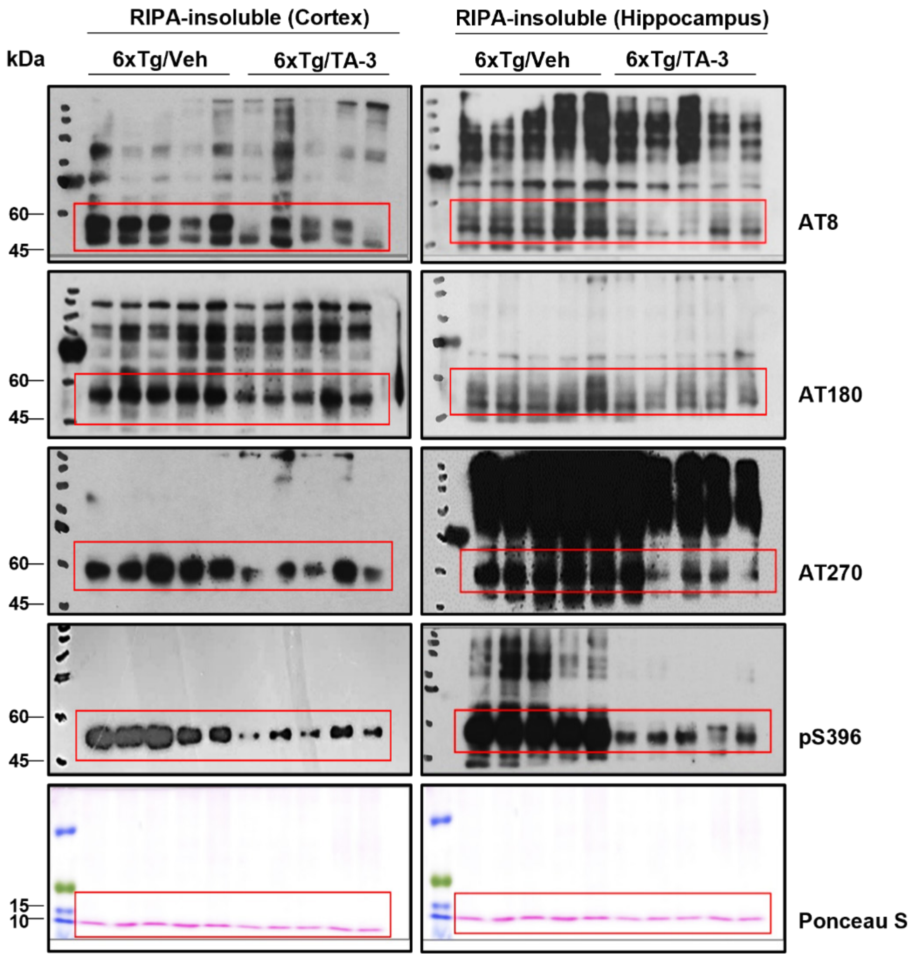

Figure S7 Full uncropped gel pictures of the RIPA-soluble and -insoluble fractions of cortical and hippocampal lysates from 6xTg mic treated with TA-3 or vehicle for two months. Both fractions were subjected to immunoblot analysis using indicated antibodies. For analysis of insoluble fraction, Ponceau S staining was employed to better visualize protein loading, and full blot images are presented to indicate sizes of the protein bands.

Table S1 Pharmacokinetic parameters after single i.p. administration of TA-3 of different doses. (mean ± SEM, *n* = 4)

	1 mg/kg	5 mg/kg	25 mg/kg
$AUC_{plasma,0\text{-}24\ h}$ (ng/mL x h)	1793.47 ± 324.42	3356.93 ± 477.07	20332.22 ± 827.47
$AUC_{ISF,0\text{-}24\ h}$ (ng/mL x h)	172.66 ± 28.36	254.98 ± 27.41	230.09 ± 31.81
$C_{max,plasma}$ (ng/mL)	199.31 ± 20.81	1312.44 ± 278.12	8845.25 ± 140.37
$C_{max,ISF}$ (ng/mL)	10.82 ± 1.18	16.47 ± 1.42	17.09 ± 3.04
$C_{last,plasma}$ (ng/mL)	14.17 ± 5.75	34.01 ± 14.05	66.56 ± 12.31
$C_{last,ISF}$ (ng/mL)	6.79 ± 1.50	10.08 ± 0.67	8.79 ± 1.55
$T_{max,plasma}$ (h)	0.67 ± 0.08	0.83 ± 0.08	2.00 ± 0.00
$T_{max,ISF}$ (h)	1.17 ± 0.17	2.00 ± 0.00	3.00 ± 0.00
$t_{1/2,plasma}$ (h)	3.72 ± 0.49	3.98 ± 0.12	4.07 ± 0.18
$t_{1/2,ISF}$ (h)	4.89 ± 0.78	5.67 ± 1.14	5.78 ± 0.55
Whole brain concentration at 24 h (ng/g)	9.08 ± 0.01	13.38 ± 0.09	35.81 ± 0.36
Plasma concentration at 24 h (ng/mL)	14.17 ± 5.75	22.35 ± 2.62	66.56 ± 12.31
B/P ratio at 24 h	0.73 ± 0.21	0.62 ± 0.07	0.58 ± 0.14

Supplementary Methods

Synthesis of TauAutac (TA)

To synthesize *tert*-Butyl (6-chloro-9H-purin-2-yl) carbamate (**2** in Figure 1A), $(Boc)_2O$ (1.5 g, 7.08 mmol) was added to a stirred solution of 2-amino-6-chloropurine (1.0 g, 5.9 mmol) in DMSO (12 mL) at 0°C, and the mixture was warmed slowly to RT. A catalytic amount of DMAP was added and the solution was stirred for 8 h. The reaction mixture was diluted with water and extracted with ethyl acetate. The organic layer was dried over Na_2SO4 and concentrated under vacuum to give **1** in Figure 1A which was used for the next step without any further purification. To a stirred solution of **1** of Figure 1A in THF (30 mL), 60% NaH (350 mg, 8.8 mmol) was added in portion at 0°C and the mixture was warmed slowly to RT. After completion of the reaction, the mixture was quenched with ice-water, extracted with ethyl acetate and washed with brine (2 mL). The organic layer was then concentrated under vacuum and the residue was purified by column chromatography (MeOH/ CH_2Cl_2) to give **2** as a white solid (1.1 g, 72% in 2 steps). ^1H NMR (300 MHz, DMSO-d_6): δ 13.60 (br, 1H), 10.22 (br, 1H), 8.45 (s, 1H), 1.43 (s, 9H); LC-MS: $[M+H]^+$ 270.1.

To synthesize *tert*-butyl (6-chloro-9-(4-fluorobenzyl)-9H-purin-2-yl) carbamate (**3** in Figure 1A), 4-fluorobenzyl alcohol (0.6 mL), triphenylphosphine (2.4 g, 9.2 mmol) was added to a stirred solution of **2** of Figure 1A (1 g, 3.7 mmol) in THF (19 mL), followed by dropwise addition of diethyl azodicarboxylate (2 mL) at RT. The reaction mixture was stirred at RT for six h for completion of the reaction. The reaction mixture was concentrated under vacuum and the residue was purified by column chromatography ($MeOH/CH_2Cl_2$) to give **3** as a yellow solid (1.0 g, 74%). ^1H NMR (300 MHz, CDCl3); LC-MS: $[M+H]^+$ 378.1.

To synthesize 2-amino-9-(4-fluorobenzyl)-1,9-dihydro-6H-purin-6-one (**4** in Figure 1A), **s**olution of **3** (1 g) in 80% formic acid solution (10.9 mL) was stirred and heated to 80°C for 2

h. The reaction mixture was concentrated under vacuum to dry it completely, and the residue was washed repeatedly with CH_2Cl_2 and 2% MeOH/CH_2Cl_2 to get ~92% pure **4** (601 mg, 65%). ^{1}H NMR (400 MHz, DMSO-d_6); LC-MS: $[M+H]^+$ 260.1.

To synthesize N-acetyl-S-(2-amino-9-(4-fluorobenzyl)-6-oxo-6,9-dihydro-1H-purin-8-yl)-L-cysteine (**6** in Figure 1A), compound **4** (900 mg) was partially dissolved in 100 mL of distilled water, and 30 mL bromine water was added slowly dropwise to the reaction mixture from a dropping funnel at RT. After stirring for 12 h at RT, the resulting precipitate was filtered, washed with water, and dried in an oven to give compound **5** in Figure 1A, which was used for the next step without further purification. Compound **5**, K_2CO_3 (3.8 g, 27 mmol) and N-acetyl-L-cysteine (NAC) (2.2 g, 14 mmol) were dissolved in DMF (15 mL), and the mixture was heated to 90°C for 5 h. The mixture was then cooled to RT, and the crude material was purified by stepwise acidic solidification. Viz., DMF was removed under vacuum, and the crude residue was dissolved in water, followed by removal of the undissolved solid by filtration. The clear filtrate was then slowly acidified to pH ~6, and, after the removal of the solid, further acidified to pH ~4. The solution was kept for some time to obtain pure white solid **6** (600 mg, 58% in 2 steps). ^{1}H NMR (400 MHz, DMSO-d_6): δ 12.88 (s, 1H), 10.63 (s, 1H), 8.51 (d, J=7.7 Hz, 1H), 7.19 (t, J=7.1, 2H), 7.16 (t, J=7.8, 2H), 6.51 (s, 2H), 5.08 (s, 1H), 5.07 (s, 1H), 4.43-4.47 (m, 1H), 3.56 (dd, J=4.6, 16.9, 1H), 3.35 (m, 1H), 1.81 (s, 3H); LC-MS: $[M+H]^+$ 421.1.

To synthesize 3,7-bis(dimethylamino)-10H-phenothiazine-10-carbonyl chloride (**7** in Figure 1B), CH_2Cl_2 (25 m) and Na2CO3 (6.63 g, 62.52 mmol) were added to a solution of methylene blue (5.00 g, 15.63 mmol) in 50 mL of water, and the mixture was stirred at 40 °C under nitrogen atmosphere. Sodium dithionite (10.89 g, 62.52 mmol) dissolved in 70 mL of water was injected directly to the solution using a syringe device. The mixture was then

stirred at 40 °C under nitrogen atmosphere until the solution turned yellow. The reaction mixture was cooled with an ice-water bath to which triphosgene (2.78 g, 9.38 mmol) in 20 mL of dichloromethane was added dropwise, followed by stirring for another 1 h. The solution was poured into 200 mL of ice-water with stirring, and the resulting mixture was extracted with 3 × 100 mL of CH_2Cl_2. The combined extracts were washed with brine, dried over Na_2SO_4, evaporated on a rotary evaporator, and then purified by column chromatography (ethyl acetate/*n*-hexane=1/10) to yield **7** as a blue solid. Yield 2.83 g, 55%. ^{1}H NMR (400 MHz, DMSO-d_6): LC-MS: $[M+H]^+$ 348.8.

To synthesize **8(1~5)** of Figure 1B, Et_3N (1.5 eq.) was added to a stirred solution of **7** of Figure 1B (1 eq.) in CH_2Cl_2 at 0°C, followed by addition of the corresponding amine (1.2 eq.) and stirring at RT for 30 min. The reaction mixture was quenched by addition of water and extracted several times with IPA/$CHCl_3$ (1/4). The combined extracts were washed with brine, dried over Na_2SO_4, and evaporated on a rotary evaporator to get **8(1~5)** as a blue solid which was used for the next step reaction without any further purification.

To synthesize TA-1~5, DIPEA was added to a stirred solution of **6** of Figure 1B (1 eq.) in DMF, followed by addition of HATU (1.5 eq.) and stirring for 10 min at RT. The corresponding amines **8(1~5)** (1.2 eq.) in DMF were then added to the reaction mixture, which was stirred for 4~6 h at RT. The reaction mixture was quenched by adding water and stirred several times to get a blue solid, which was filtered, dried in oven and purified by column chromatography (MeOH/ CH_2Cl_2) to give pure **TA-1~5**.

TA-1: (*R*)-N-(3-(2-Acetamido-3-((2-amino-9-(4-fluorobenzyl)-6-oxo-6,9-dihydro-1H-purin-8-yl)thio)propanamido)propyl)-3,7-bis(dimethylamino)-10H-phenothiazine-10-carboxamide. ^{1}H NMR (400 MHz, DMSO-d_6) δ 11.37 (s, 1H), 10.64 (s, 1H), 8.44 (d, *J*=8.0 Hz, 1H), 8.03

(t, *J*=5.5 Hz, 1H), 7.33-7.24 (m, 2H), 7.23-7.11 (m, 4 H), 6.73-6.62 (m, 4H), 6.54 (brs, 2H), 6.10 (t, *J*=5.7 Hz, 1H), 5.07 (s, 1H), 4.54-4.36 (m, 1 H), 3.18-2.98 (m, 6H), 2.88 (s, 12H), 1.85 (s, 3H), 1.58-1.44 (m, 2H); ^{13}C NMR (101 MHz, DMSO-d$_6$) δ 169.8, 163.0, 160.7, 156.1, 155.7, 154.0, 153.2, 148.7, 142.5, 133.5, 132.8, 129.5, 129.4, 128.8, 127.7, 117.0, 116.1, 115.8, 111.6, 110.7, 54.0, 45.1, 42.3, 40.7, 36.5, 30.0, 23.0; LC-MS: [M+H]$^+$ 788.9 ; HRMS (ESI): calcd. for $C_{37}H_{42}FN_{12}O_4S_2$ [M + H]$^+$ 788.2847; found 788.2897.

TA-2: (*R*)-N-(4-(2-Acetamido-3-((2-amino-9-(4-fluorobenzyl)-6-oxo-6,9-dihydro-1H-purin-8-yl)thio)propanamido)butyl)-3,7-bis(dimethylamino)-10H-phenothiazine-10-carboxamide. ^{1}H NMR (400 MHz, DMSO-d$_6$) δ 10.71 (brs, 1H), 8.40 (d, *J*=8.1 Hz, 1H), 8.04 (t, *J*=5.5 Hz, 1H), 7.31-7.09 (m, 6H), 6.73-6.56 (m, 5H), 5.99 (t, *J*=5.6 Hz, 1H), 5.07 (s, 2H), 4.47 (dd, *J*= 13.4, 8.0 Hz, 1H), 3.54-3.36 (m, 1H), 3.31-3.20 (m, 1H), 3.11-2.94 (m, 4H), 2.88 (s, 12H), 1.85 (s, 3H), 1.50-1.28 (m, 4H); ^{13}C NMR (75 MHz, DMSO-d$_6$) δ 169.9, 163.6, 160.3, 156.2, 155.7, 154.2, 153.2, 148.9, 142.6, 133.6, 133.0, 129.5, 129.4, 128.7, 127.8, 117.1, 116.1, 115.8, 111.6, 110.7, 52.8, 45.1, 40.7, 35.2, 27.5, 26.8, 23.0; LC-MS: [M+H]$^+$ 802.9; HRMS (ESI): calcd. for $C_{38}H_{44}FN_{12}O_4S_2$ [M + H]$^+$ 802.3003; found 788. 802.3061.

TA-3: (R)-N-(2-(2-(2-Acetamido-3-((2-amino-9-(4-fluorobenzyl)-6-oxo-6,9-dihydro-1H-purin-8-yl)thio)propanamido)ethoxy)ethyl)-3,7-bis(dimethylamino)-10H-phenothiazine-10-carboxamide. ^{1}H NMR (400 MHz, DMSO-d$_6$) δ 10.64 (s, 1H), 8.41 (d, *J*=8.0 Hz, 1H), 8.08 (t, *J*=5.5 Hz, 1H), 7.31-7.10 (m, 6H), 6.72-6.61 (m, 4H), 6.53 (brs, 2H), 5.97 (t, *J*=5.5 Hz, 1H), 5.07 (s, 2H), 4.49 (td, *J*=8.1, 5.4 Hz, 1H), 3.44-3.35 (m, 5H), 3.23-3.13 (m, 5H), 2.88 (s, 12H), 1.84 (s, 3H). ^{13}C NMR (101 MHz, DMSO-d$_6$) δ 170.2, 169.9, 163.2, 160.9, 156.1, 155.6, 154.1, 153.2, 148.9, 142.7, 133.5, 133.0, 129.5, 129.4, 128.7, 127.7, 117.0, 116.0, 115.8, 111.6, 110.7, 69.5, 69.1, 52.8, 44.8, 40.7, 39.1, 35.1, 23.0; LC-MS: [M+H]$^+$ 818.9.

HRMS (ESI): calcd. for $C_{38}H_{44}FN_{12}O_5S_2$ [M + H]$^+$ 818.2952; found 818.3006.

TA-4: (*R*)-N-(4-(2-Acetamido-3-((2-amino-9-(4-fluorobenzyl)-6-oxo-6,9-dihydro-1H-purin-8-yl)thio)propanamido)cyclohexyl)-3,7-bis(dimethylamino)-10H-phenothiazine-10-carboxamide. ^{1}H NMR (400 MHz, DMSO-d_6) δ 10.61 (s, 1H), 8.32 (d, *J*=8.1 Hz, 1H), 7.93-7.82 (m, 1H), 7.34-7.27 (m, 2H), 7.24-7.11 (m, 4H), 6.74-6.60 (m, 4H), 6.55 (brs, 2H), 5.28 (d, *J*=7.3 Hz, 1H), 5.09 (s, 2H), 4.57-4.39 (m, 1H), 3.78-3.63 (m, 1H), 3.62-3.52 (m, 2H), 2.87 (s, 12H), 1.82 (s, 3H), 1.63-1.41 (m, 8H); ^{13}C NMR (101 MHz, DMSO-d_6) δ 169.6, 169.0, 160.6, 155.9, 154.4, 153.9, 152.8, 148.5, 142.3, 133.4, 133.0, 129.4, 129.3, 128.4, 127.0, 117.0, 115.9, 115.8, 111.3, 110.7, 52.3, 44.9, 40.6, 35.3, 28.5, 28.4, 28.0, 22.8; LC-MS: [M+H]$^+$ 829.0; HRMS (ESI): calcd. for $C_{40}H_{46}FN_{12}O_4S_2$ [M + H]$^+$ 828.3160; found 828.3212.

TA-5: (R)-N-(3-((2-amino-9-(4-fluorobenzyl)-6-oxo-6,9-dihydro-1H-purin-8-yl)thio)-1-(4-(3,7-bis(dimethylamino)-10H-phenothiazine-10-carbonyl)piperazin-1-yl)-1-oxopropan-2-yl)acetamide. ^{1}H NMR (400 MHz, DMSO-d_6) δ 10.64 (s, 1H), 8.37 (d, *J*=8.5 Hz, 1H), 7.51 (d, *J*=8.9 Hz, 2H), 7.25-7.10 (m, 3H), 6.76-6.60 (m, 4H), 6.53 (brs, 2H), 5.04 (s, 2H), 5.03-4.91 (m, 1H), 3.66-3.54 (m, 2H), 3.46-3.35 (m, 2H), 3.30-3.23 (m, 2H), 3.20-3.05 (m, 4H), 2.87 (s, 12H), 1.76 (s, 3H); ^{13}C NMR (75 MHz, DMSO-d_6) δ 169.3, 168.7, 162.5, 155.9, 155.1, 154.1, 153.2, 151.7, 142.6, 133.0, 132.9, 129.5, 129.4, 124.5, 116.9, 116.0, 115.7, 110.9, 53.9, 48.3, 44.9, 42.2, 29.1, 28.0, 22.5; LC-MS: [M+H]$^+$ 801.0; HRMS (ESI): calcd. for $C_{38}H_{42}FN_{12}O_4S_2$ [M + H]$^+$ 799.2847; found 799.2814.

In vivo pharmacokinetic study

To assessment of microdialysis probe recovery *in vivo*, a total of 6 kDa CMA 7 microdialysis

probes (Harvard Apparatus, Holliston, MA, USA) were connected with PE/PVC tubing (0.6 x 1.6 mm) to a PHD ULTRA^{TM} syringe pump (Harvard apparatus, Holliston, MA, USA). For equilibration of microdialysis probe, the inlet of the microdialysis probe was perfused with filtered artificial cerebrospinal fluid (aCSF) buffer containing 122 mM NaCl, 1.3 mM $CaCl_2$, 1.2 mM $MgCl_2$, 3.0 mM KH_2PO_4, and 25.0 mM $NaHCO_3$ at a flow rate of 0.5 μL/min, and the outlet was kept in an empty E-P tube for 1 h, with the membrane of the microdialysis probe soaked in ethanol. To estimate *in vivo* recovery, retrodialysis was performed with aCSF containing TA-3. The *in vivo* recovery was calculated as equivalent to the loss upon retrodialysis using the following equation: *In vivo* recovery = $(C_{perfusate} - C_{diaysate})/C_{perfusate}$ where $C_{perfusate}$ is the TA-3 concentration in the perfusate (inlet of the microdialysis probe), and $C_{diaysate}$ is the TA-3 concentration in the dialysate (outlet of the microdialysis probe). Recovery was utilized to calculate the concentration of the TA-3 in the brain interstitial fluid (ISF) (C_{ISF}) from the measured brain ISF dialysate concentration.

To implant a microdialysis guide cannula, five-week-old male BALB/c mice (20~25 g) were positioned on a stereotactic device (Harvard Apparatus, USA) using an ear bar under 1–1.5% isoflurane anesthesia in N_2O:O_2 (70:30 vol%). A guide cannula was inserted into the right hippocampus [-2.0 mm anterior/posterior (AP), 1.5 mm medial/lateral (ML), and -2.0 mm dorsal/ventral from the bregma] after drilling a small hole on the skull. The guide cannula was fixed with dental cement, and the skin wound was sealed with sutures. Animals were then returned to their cages for one day, and allowed to recover from the surgery.

For stabilization prior to compound administration, microdialysis probes were perfused with aCSF at a flow rate of 0.5 μL/min for one h with the membrane of the microdialysis probe soaked in ethanol. Male BALB/c mice (20-30 g) were given TA-3 at the dose of 1,

5 and 25 mg/kg by i.p. administration of aqueous solution (100 μL of 1 mg/mL in PBS with 5% DMSO and 5% Tween 80). Perfusates from the microdialysis probes were collected every 30 min for four h after administration, at eight and 24 h. Dialysate samples were stored at -80 °C until LC-MS/MS analysis. Blood samples (30 μL) were collected from the saphenous vein at 2, 5, 10, 15, 30, 45, 60, 120, 240, 480 and 1,440 min after administration. Blood samples were immediately centrifuged at 12,000 x *g* for 10 min at 4 °C to recover plasma. The brain was then excised 24 h post-administration, and homogenized using Bioprep-24 homogenizer (Bioand, Seoul, Korea) after adding PBS equivalent to the weight of each tissue sample. Plasma and homogenized brain tissue samples were stored at -80 °C until LC-MS/MS analysis. Plasma and dialysate concentrations were determined by LC-MS/MS, and pharmacokinetic parameters were estimated by non-compartmental analysis using WinNonlin 2.1 (Pharsight, Mountain View, CA). The whole brain concentration without interference from blood was determined by subtracting the drug levels in brain plasma (plasma volume x plasma concentration) from the measured drug levels in brain homogenates [1].

Sample preparation for pharmacokinetic study

The compounds were isolated from plasma, brain microdialysate and whole-brain homogenat es by liquid-liquid extraction. Plasma (10 μL), brain microdialysate (10 μL) or brain homoge nate samples (50 μL) were mixed with 10 μL of the mobile phase containing an internal stand ard by vortexing for 1 min. Then, 250 μL ethyl acetate was added to the mixture and vortexed for 1 min. After centrifugation at 10,000 rpm, 4 °C for 10 min, organic layer was separated an d evaporated to dryness under vacuum. Finally, the dried residue was reconstituted in 20 μL o f mobile phase (acetonitrile and 0.01% formic acid-water, 50:50%, v/v) for immediate analysi

s by LC-MS/MS.

LC-MS/MS

The LC-MS/MS system consisted of an Agilent LC 1100 series (Agilent Technologies, CA, USA) binary pump, vacuum degasser and auto-sampler system connected to a 6490 triple quadrupole MS equipped with an electrospray ionization (ESI) source Agilent jet stream technology. Chromatographic separation was achieved using an analytical Agilent ZORBAX Extend-C18 (120 Å 1.0 × 150 mm, 3.5 micron) column. The column temperature was maintained at 30 °C. The temperature of the auto-sampler was set at 4 °C. Two µL of sample solutions were injected, and the analytes were eluted using of acetonitrile and 0.1% formic acid in water (80:20, v/v) pumped at a constant flow rate of 0.1 mL/min. The isocratic separation run time was 5 min. The MS/MS system was operated under positive ESI, and the multiple reactions monitoring (MRM) mode was used to identify the compounds of interest. The MS operational parameters were: argon as a collision gas; capillary voltage of 5 kV; gas temperature of 225 °C; gas flow of 15.1 l/min; and collision energies of 30 eV. Detection of the analytes was performed using the MRM mode to monitor the precursor to product ion transitions of 818.2-286.1 *m/z* for TA-3.

Calculation of lysosomal dysfunction index and lysosomal degradation efficiency index

If we assume that the production rate P of an autophagic substrate or target is constant and un affected by lysosomal stressors and that the current degradation rate of the substrate is propor tional to the current level of the substrate (as reflected by the band intensity in immunoblots) with a coefficient of k which is constant throughout the study, the time evolution of the substr

ate level y, is governed by

$\frac{dy}{dt} = P - ky$ (1)

Dividing both sides by P – ky, and integrating them, we obtain

$-\frac{1}{k}\ln(P - ky) = t + C$ or $P - ky = e^{-k(t+C)}$ (2)

where the integration constant C is to be determined by the initial condition. Imposing the initial condition $y = 0$ at $t = 0$ leads to $e^{-kC} = P$, which in turn yields

$P - ky = P * e^{-kt}$ and $y = \frac{P}{k}(1 - e^{-kt})$ (3)

Specifically, the normal substrate level y_1 and the lysosomal stressed substrate level y_2 are given by

$y_1 = \frac{p}{k1}(1 - e^{-k1t})$ and $y_2 = \frac{p}{k2}(1 - e^{-k2t})$ (4)

where k_1 and k_2 are the corresponding degradation coefficients.

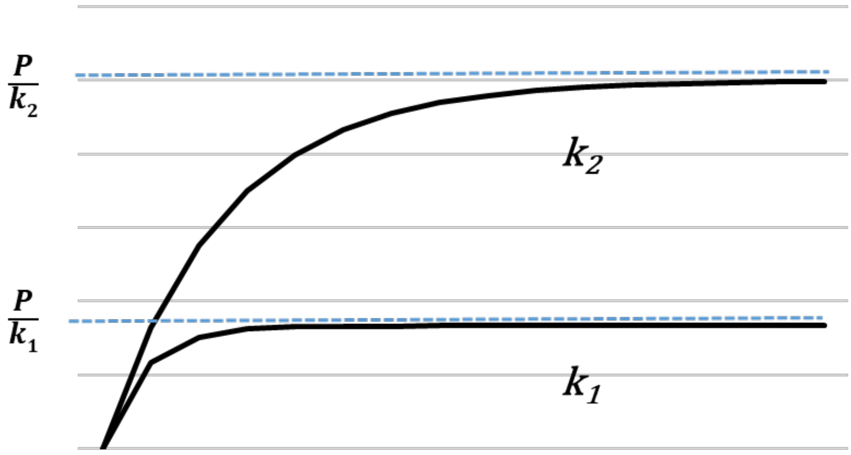

When sufficient time is allowed ($t \to \infty$), the substrate level approaches the asymptotic

value $y_i(t \to \infty) \equiv y_{i\infty} = P/k_i$ for $i = 1$ and 2, resulting in the lysosomal dysfunction index

$$\frac{y_{2\infty}}{y_{1\infty}} = \frac{k_1}{k_2} \quad (5)$$

The reciprocal of the lysosomal dysfunction index could be regarded as an index representing lysosomal degradation efficiency (lysosomal degradation efficiency index).

$$\frac{y_{1\infty}}{y_{2\infty}} = \frac{k2}{k1} \quad (6)$$

In the real experiment studying the effect of doxycycline-induced Tau expression, basal LC3-II level is not 0 and LC3-II increased by doxycycline treatment is superimposed on the basal LC3-II level without doxycycline. Therefore, the initial value of y_2 is not zero but given by the asymptotic value of y_1: $y_2(t = 0) = y_{1\infty} = P/k_1$. Applying this to Eq. 2 at $t = 0$, we have

$$P - k_2\frac{P}{k_1} = e^{-k_2C} \quad (7)$$

Thus,

$$P - k_2y_2 = e^{-k_2t}\left(P - k_2\frac{P}{k_1}\right) = P * \left(\frac{k1-k2}{k1}\right) * e^{-k2t} \quad (8)$$

which yields

$$y2 = \frac{\mathrm{p}}{k2}\ (1 - \frac{k1-k2}{k1}e^{-k2t}\ ) \quad (9)$$

In the asymptotic limit ($t \to \infty$), we still

$$y_{2\infty} = \frac{\mathrm{p}}{k2} \quad (10)$$

and thus again

$$\frac{y_{2\infty}}{y_{1\infty}} = \frac{k_1}{k_2} \quad (11)$$

which is the same as Eq. (5). Thus, the lysosomal stress index is given only by the ratio of the degradation coefficients, regardless of the basal LC3-II level at the time of doxycycline additi on.

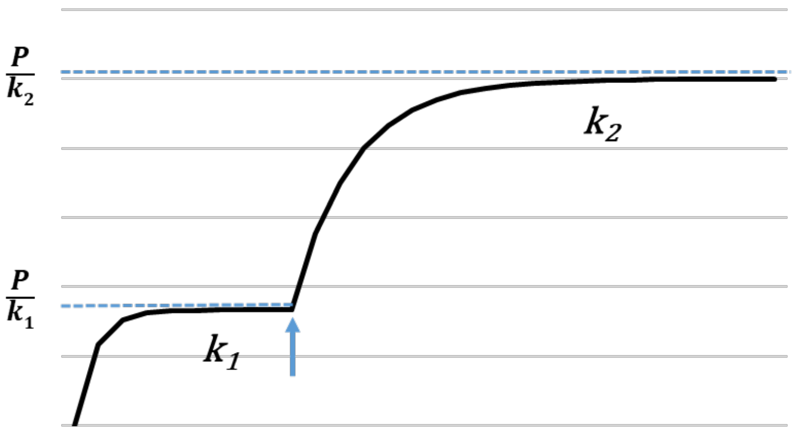

We considered the treatment period of T-REx-Tau40-HEK293 cells with doxycycline for ≥ 72 h to be long enough to calculate the asymptotic values of the lysosomal stress index sinc e the LC3-II level reached a plateau after 72 h of incubation without affecting the cell conditi on (See Figure S5A).

Supplementary References

1. Fischer D, Osburg B, Petersen H, Kissel T, Bickel U. Effect of poly(ethylene imine) molecular weight and pegylation on organ distribution and pharmacokinetics of polyplexes

with oligodeoxynucleotides in mice. Drug Metab Dispos. 2004; 32:983-992.
